# Pilot, double-blind, randomized, placebo-controlled clinical trial of the supplement food Nyaditum resae^®^ in adults with or without latent TB infection: Safety and immunogenicity

**DOI:** 10.1371/journal.pone.0171294

**Published:** 2017-02-09

**Authors:** Eva Montané, Ana Maria Barriocanal, Ana Lucía Arellano, Angelica Valderrama, Yolanda Sanz, Nuria Perez-Alvarez, Paula Cardona, Cristina Vilaplana, Pere-Joan Cardona

**Affiliations:** 1 Department of Clinical Pharmacology, Hospital Universitari Germans Trias i Pujol, Badalona, Catalonia, Spain; 2 Department of Pharmacology, Therapeutics and Toxicology, Universitat Autònoma de Barcelona, Catalonia, Spain; 3 Fundació Institut Germans Trias i Pujol, Badalona, Catalonia, Spain; 4 Lluita Contra la Sida Foundation, Badalona, Catalonia, Spain; 5 Statistics and Operations Research Department, Universitat Politècnica de Catalunya- BarcelonaTech, Barcelona, Catalonia, Spain; 6 Unitat de Tuberculosi Experimental, Universitat Autònoma de Barcelona, CIBERES, Fundació Institut Germans Trias i Pujol, Badalona, Catalonia, Spain; National Institute for Infectious Diseases (L. Spallanzani), ITALY

## Abstract

**Background:**

Nyaditum resae^®^ (NR) is a galenic preparation of heat-killed *Mycobacterium manresensis*, a new species of the *fortuitum* complex, that is found in drinkable water, and that has demonstrated to protect against the development of active TB in a murine experimental model that develop human-like lesions.

**Methods:**

Double-blind, randomized, placebo-controlled Clinical Trial (51 volunteers included). Two different doses of NR and a placebo were tested, the randomization was stratified by Latent Tuberculosis Infection (LTBI)-positive (n = 21) and LTBI-negative subjects (n = 30). Each subject received 14 drinkable daily doses for 2 weeks.

**Results:**

All patients completed the study. The 46.3% of the overall reported adverse events (AE) were considered related to the investigational treatment. None of them were severe (94% were mild and 6% moderate). No statistical differences were found when comparing the median number of AE between the placebo group and both treatment groups. The most common AE reported were gastrointestinal events, most frequently mild abdominal pain and increase in stool frequency. Regarding the immunogenic response, both LTBI-negative and LTBI-positive volunteers treated with NR experienced a global increase on the Treg response, showed both in the population of CD25+CD39-, mainly effector Treg cells, or CD25+CD39+ memory PPD-specific Treg cells.

**Conclusion:**

This clinical trial demonstrates an excellent tolerability profile of NR linked to a significant increase in the population of specific effector and memory Tregs in the groups treated with NR in both LTBI-positive and negative subjects. NR shows a promising profile to be used to reduce the risk of active TB.

## 1 Introduction

Tuberculosis is one of the most frequent infectious diseases in the world, in spite of being a curable disease [1]. Even if a vaccine (Bacille Calmette Guérin -BCG-) has been available since 1927 and extensively used (3 billion doses used), there is a consensus that it only helps to stop the development of disseminated and meningeal TB [2]. In fact it has been experimentally demonstrated that BCG vaccination stops the bacillary growth in vaccinated subjects sooner than in the unvaccinated, but does not avoid the infection by *Mycobacterium tuberculosis* (Mtb) [3].

One of the characteristics of Mtb infection, is that it is transmitted through aerosol and that disease is not usually the immediate consequence of the infection [4], resulting in LTBI, were the bacilli is able to remain in the subjects for a long period, even years. It has been estimated that approximately a third of mankind already has LTBI [5] without hindering the host. Lesions of 1 mm in diameter at the lung parenchyma are irrelevant to challenge normal lung function [6], which is why most of them ignore the fact of being infected. The only way to know that someone has LTBI is by performing the tuberculin skin test (TST) or the T-cell interferon-gamma release assays (TIGRA) [7]. A low percentage (around 10%) will develop active TB (TB) [5], and besides severe immunodeficiencies (like AIDS) [1], the mechanism of other comorbid factors are poorly known.

A new theory supported by experimental data and published recently suggests that the development of active TB is caused by an exaggerated response against Mtb. This response is based on a progressive massive neutrophilic infiltration of the lesions (causing a sudden increase in size) and with the presence of nearby coincident lesions; a process favoured by the environment of the upper lobes, that leads to the coalescence of the lesions [8,9]. This mechanism could also explain why subjects with diabetes mellitus (DM) have 3 times more risk to develop TB [10,11]. DM is a consequence of an imbalance in the Treg/Th17 response [12], generating a global exaggerated inflammatory response.

An excessive inflammatory response in LTBI would be materialized through a neutrophilic infiltration fuelled by Th17 cells, stimulated by the Mtb infection itself in an intensity that depends on the host reactivity [13]. It has been demonstrated that in subjects with LTBI there is an inverse relation between the Th17 and Treg cells [14]; and that Th17 response can be counterbalanced by the presence of Tregs [15–17]. In this context, we decided to induce a Treg response through a low dose tolerance process using heat-killed Mtb (HKMtb) cells, and several environmental mycobacteria for their cross-immunity with Mtb [18], with successful results. Among the environmental mycobacteria tested, a species usually found in drinking water and thus able to be considered as a food supplement [19] was isolated. It was identified to be part of the *fortuitum* complex, according to a state-of-the-art multiplex probe assay [20] which is the standard for mycobacterium identification in clinical laboratories. After its sequencing, it was considered a new species, called *M*. *manresensis* (Certificate of Deposit CECT 8638 at the Spanish Type Culture Collection) [21]. This strain has been chosen to be further developed as food supplement for its similar ability to induce the protection obtained with HKMtb [22].

Oral administration of alive or heat-killed *M*. *manresensis* was found to stop the inflammatory progression towards TB in an experimental murine model, a process linked to the increase of PPD-specific memory Tregs (CD25+CD39+) [22]. This new tool that decreases the risk of progression towards TB was patented by the Institut d’Investigació Germans Trias i Pujol (IGTP) (PCT/ES2013/000145) and transferred to a spin-off of the same Institute (Manremyc sl) for its development.

The study presented here is a pilot, double-blind, randomised, masked and placebo controlled clinical trial (CT), conducted in volunteers, to evaluate the tolerability and the immunogenicity of 2 oral doses of Nyaditum resae^®^ (a preparation of heat-killed *M*. *manresensis*) administered daily for 14 days to the general population, both LTBI positive or negative.

## 2 Materials and methods

### 2.1 Ethics

The protocol of the study was reviewed and approved by the Ethics Committee at the investigational centre (Hospital Universitari Germans Trias i Pujol, Badalona, Catalonia, Spain). All investigators and collaborators agreed to rigorously observe the Helsinki declaration with all its amendments and to follow the Good Clinical Practice guidelines of the ICH (International Conference on Harmonisation of Technical Requirements for Registration of Pharmaceuticals for Human Use). The objectives and methodology, as well as possible drawbacks and risks due to the study, were explained to each subject orally and in writing (Subject Information Sheet) before their inclusion. They were also informed of the different treatments to be tested, the way they would be assigned to the groups, the option to withdrawal from the study at any time and of the existence of an insurance contract. Informed consent was obtained of all the participating volunteers by consent form signature before starting any study procedure. Participants were also informed and signed their consent to be included in a local register from the Health Department of the Catalan Government to control the participation of healthy volunteers in Phase I clinical trials. The trial has been registered in ClinicalTrials.gov: NCT02076139.

### 2.2 Participants

Subjects were interviewed and screened for enrolment at the Phase I Unit of the Hospital Germans Trias i Pujol (Badalona, Catalonia, Spain) by the clinical pharmacologists. The target population of the study was healthy adults, with or without LTBI. A screening visit was made for each volunteer where a complete anamnesis and physical exam, laboratory parameters tests (hemato-biochemical and immunogenicity parameters), Tuberculin Skin Test (TST), serology against HIV and a pregnancy test were performed. Previous TST was acceptable and was not repeated when positive in the last 5 years or negative in the last 6 months. Radiological chest X-ray exam was performed in TST-positive volunteers. Subjects were not included in the study if any of the following criteria were present: TB, immunodeficiencies, chronic immunosuppressant therapy, reception of blood products or derivatives six months prior randomization, pregnancy or lactation.

Furthermore, the investigator’s team certifies that to their knowledge all subjects were considered able to be included in the CT, in terms of fulfilling all the study’s requirements.

### 2.3 Interventions

**Fig 1:**
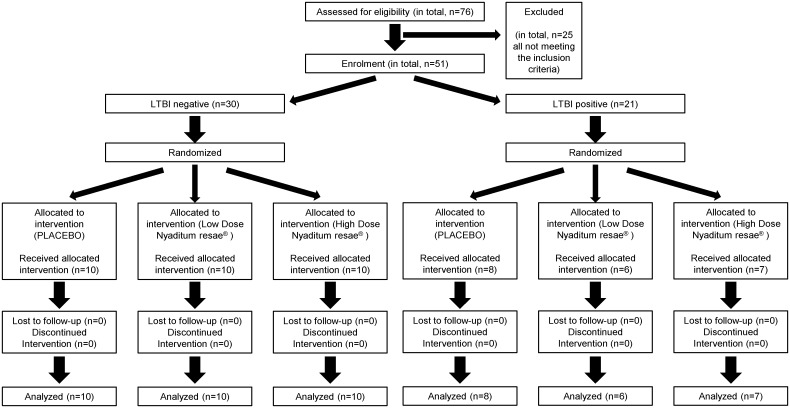
Chart representing the participant flow of the CT. From 76 people interviewed for eligibility, 51 could be included. All the 51 were randomized and allocated to treatment, receiving it, being fully followed-up, and ending the trial and being analysed.

After the inclusion period, the volunteers were randomized in a proportion 1:1:1 to receive either placebo or the Nyaditum resae^®^ (NR) in low (10^4^) or high (10^5^) doses, stratified by TST status (Fig 1). The trial was originally designed to allocate 10 subjects per treatment group and TST status. Once the groups with TST-negative were fulfilled, the screening process was focused in looking for TST-positive subjects. NR was supplied by the sponsor (Manremyc S.L., Manresa, Catalonia, Spain) as a preparation of drinkable vials containing heat-killed *Mycobacterium manresensis* dissolved in distilled water. NR was produced under Good Manufacturing Practice by the Laboratory Reig Jofre (Sant Joan Despí, Catalonia, Spain). Placebo was identically supplied and formulated except that it only contained distilled water. Only a single batch of both NR and placebo were used in the whole study. The distribution of the treatment vials for the CT was carried out by the Clinical Research Organisation (CRO) of the study, Fundació per la Lluita contra la Sida -FLS Research Support- (Badalona, Catalonia, Spain), where the entire study treatment received was inventoried and accounted for throughout the study.

Once randomized, volunteers received a box with 14 drinkable vials with one of the 2 doses of NR or Placebo at the Phase I Unit of the Clinical Pharmacology Department, supervised by the Investigators and following the random plan specified in the study protocol. They drank one vial every day at breakfast during 2 weeks.

Every subject included in the trial had the right to withdraw at any moment by immediately contacting the investigators to inform them, without any obligation to provide reasons. This participant was supposed to be replaced by another, except in the case the trial had to be ended because of safety issues, and the new participant would have received the corresponding treatment of the subject he or she replaced. If a participant was withdrawn because of an adverse event, he or she would have been followed-up by the Investigators until resolution or reaching a clinically stable endpoint. The investigators had the right to decide to terminate or suspend the trial without prior agreement of the sponsor, by promptly informing the trial subjects (assuring them appropriate therapy and follow-up), the sponsor and the regulatory authority.

### 2.4 Objectives

This trial aimed to evaluate 1) the global tolerability of the probiotic Nyaditum resae^®^ and 2) the effect on specific memory regulatory T cells (Treg) cells population.

### 2.5 Outcomes

After the administration of the first drinkable vial, each volunteer was followed-up during 6 weeks. The monitoring plan (Table 1) shows every procedure of the follow-up planned during the study.

**Table 1:** Chronogram and monitoring plan.

	Selection		Pre administration	Administration	Follow-up		
Visit	Selection		Baseline		w1	w2	w6
**Day**	-28	-25	0		7	15	42
**Week**	-4	-4	0		1	2	6
**Window (days)**	±14		0		±3	±3	±3
**Subject Information,** **Informed consent**	X						
**Inclusion/Exclusion criteria**	X		X				
**Medical History**	X						
**HIV test**	X						
**Tuberculin Skin Test (TST)**	X^A^	X^B^					
**Chest X ray**		X^C^					
**Pregnancy test in serum**	X		X				X
**Randomization**			X				
**Treatment administration**				X			
**Physical exam and vital signs**	X		X		X	X	X
**Biochemistry & Hematology**	X		X^D^		X	X	X
**Immunogenicity sample**			X		X	X	X
Volunteer diary*				X	X	X	X^E^
**Concomitant medication**	X		X		X	X	X
**Adverse events**	X			X	X	X	X

^A^ Previous TST was acceptable and not repeated when positive in the last 5 years or negative in the last 6 months.

^B^ TST readout.

^C^ In the case of Positive TST.

^D^ Not repeated if selection’s analysis performed in the previous 15 days.

^E^ Collection of Volunteer’s Diary.

* Volunteer’s Diary records every day: day and hour of the treatment intake; stool deposition (number/day) and aspect (soft/hard); nausea (degree 1 to 3); vomits (degree 1 to 3); abdominal pain (degree 1 to 3); other adverse events (degree 1 to 3). Degree 1: mild; degree 2: moderate; degree 3: severe.

The volunteers were monitored by the investigators team of the Phase I Unit in order to perform the following safety evaluations: 1) record of adverse events, both gastrointestinal and non-gastrointestinal, being reported by the subject either spontaneously or after questioning or looking at the Volunteer’s Diary, during the first 4 weeks of the monitoring (Table 1); 2) vital constants; 3) physical examination; 4) laboratory safety and immunogenicity tests.

AE detected by the investigator through interrogation or reported by the subject during the defined period of collection were recorded. AE was considered as any unwanted medical event in a subject in a clinical trial, regardless of its relationship with the intervention under evaluation. A serious AE was defined as cause of death, life threatening, requiring inpatient hospitalization, producing disability or incapacity persistent or significant or threatening the patient. The investigators determined the relationship between the study treatment and the AE as ‘not related’, ‘unlikely’, ‘possibly’, ‘probably’, and ‘definite’ according to a predefined algorithm based on the modified Karch and Lasagna algorithm used by the Spanish Pharmacovigilance System [23,24] and after a consensus reached between the clinical pharmacologists study investigators. Laboratory abnormalities were graded following the Toxicity Grading Scale Guidance provided by the Food and Drug Administration (FDA) [25]. The main safety analyses have been focused on ‘possibly’, ‘probably’, and ‘definite’ classified AE.

Blood was extracted from the volunteers by the nurse of the Phase I Unit, under fasting conditions and before any other procedure was done, at the time-points indicated in the chronogram (Table 1). The samples were properly labelled and sent to the hospital laboratories for laboratory safety testing (15 mL) and to the Experimental Tuberculosis Unit (UTE) (8 mL) for the immunogenicity testing as soon as possible. The Departments involved in the safety laboratory results (Haematology, Clinical Analysis and Microbiology) as well as the UTE, are accredited by ISO 9001 procedures. The CRO of the study (FLS Research Support) monitored all the study in order to ensure the use of standard terminology and the collection of accurate, consistent, complete and reliable data.

Blood samples were tested for circulating cell counts (erythrocytes, leucocytes and platelets), Hb, haematocrit, fasting glucose, aspartate aminotransferase, alanine aminotransferase, g-glutamyl transpeptidase, alkaline phosphatase, total bilirubin, direct and indirect bilirubin, urea N, creatinine, glomerular filtration rate, Na and K.

The reliability of the Immunogenicity data was ensured by the performance of all assays always made by the same technician (previously trained during a 6 month period) and under supervision. Accuracy of the performance of the techniques as well as the values obtained were ensured by following strict specific procedures (Standard Operating Procedures SOPs) that were previously designed and set up specifically for this CT and always under blinding premises. The sponsor also used an external auditing to review the following issues: data management and statistics, both the UTE and the Phase I Unit, source data / source documents and the Investigator File and the Trial Master File.

**Fig 2:**
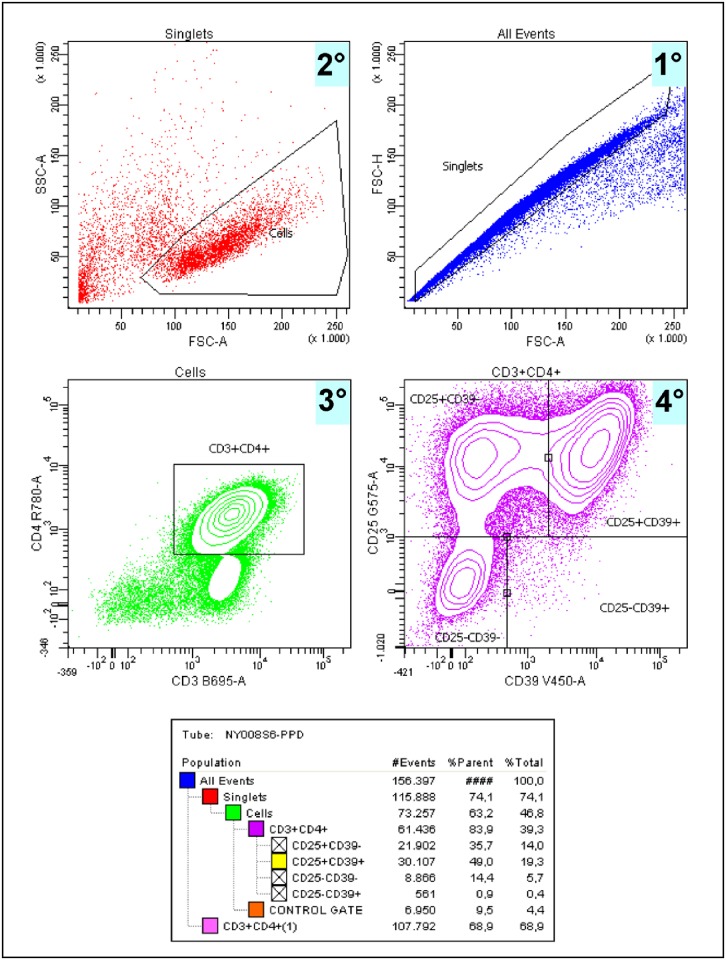
FACS analysis strategy used allowed us to find 4 different types of populations. CD25+CD39-: mainly effector Tregs (80–90%) [29]; CD25+CD39+: memory Tregs [26]; CD25-CD39-: Non Treg Effector cells; CD25-CD39+: ex-Treg cells mainly IL-17 producers [26].

Measure of specific Tregs was done according to previous studies [14,26–28]. Peripheral blood mononuclear cells (PBMC) were isolated in cell preparation tubes (CPT Becton Dickinson, BD) with sodium citrate according to the manufacturer proceedings, and processed immediately. Protein Purified Derivative (PPD) batch 49 (Statens Serum Institute, Copenhagen, Denmark) was used alone at a final concentration of 10 μg/mL to measure antigen specific responses. 1 x 10^6^ cells/well were non-stimulated or PPD-stimulated in a complete medium (RPMI-L-Gln, 10% heat-inactivated FCS, 10 U/ml penicillin, 10 U/ml streptomycin, 1 mM sodium pyruvate, 0.025 mM 2-ME) for 7 days at 37°C and 5% of CO_2_. The phenotypic analysis was performed by flow cytometry, using the following antibodies for membrane staining: PerCP-Cy 5.5 mouse anti-human CD3, APC-H7 mouse anti-human CD4, PE mouse anti-human CD25 (BD Biosciences) and Brilliant Violet 421 mouse anti-human CD39 (BioLegend). Data acquisition was performed using an LSRFortessa flow cytometer (BD Biosciences) and analysed with FACSDiva software (BD Biosciences) (Fig 2).

None of the Immunogenicity assessments performed in the present CT were standard procedures. As no cut-off exists for those non-validated and research-only techniques, a statistical difference (p<0.05) between the time-points among the same treatment group has been considered enough to prove the reactivity of the treatment.

### 2.6 Sample size

Given that this clinical trial was an exploratory study of the first administration in humans, basically intending to demonstrate the safety of the probiotic, a formal predetermination of sample size based on numeric or statistic criteria was not made. In any case, the numbers shuffled in the trial (51 subjects, 18 placebo and 33 of treatment) were within the standard for this type of studies.

### 2.7 Randomization and blinding

A random allocation scheme in which each participant has equal likelihood of being assigned to treatment versus placebo groups (ratio 1:1:1) was used. The CRO (FLS Research Support) generated an allocation list by blocks using numbers drawn from the uniform distribution. Allocation was performed considering the stratification for TST-positive and TST-negative.

Treatment is masked, meaning that neither the investigator nor the volunteer knows if the vial contains NR or Placebo. Both treatments were physically identical with the same excipients. Labelling was also identical, indicating the study code, the numerical identification of the subject and the data referred to the promoter, administration route and posology.

In an emergency case that would require to open the double blind, the researcher had to contact to the CRO which would inform the composition of the treatment of the subject. The investigator had to write a justification explaining the reasons for opening the double blind.

Investigational treatment kits were conserved in UTE and transferred to the Phase I Unit until their use. Both places performed temperature log on a daily basis.

### 2.8 Statistical methods

Results were expressed as median with the interquartile range (IQR), or as otherwise specified. All the analyses corresponding to the demographic variables, basal characteristics, as well as the variables to evaluate the Immunogenicity were made with the population per protocol (all randomized subjects who met the selection criteria, received the study treatment, and did not present major protocol deviations). All the analyses corresponding to the security variables were carried out in the safety population (all participants who received at least ten doses of the treatment).

Continuous variables were compared using non parametrical tests, Mann-Whitney and Kruskal-Wallis for non-paired and Wilcoxon test for paired data. The Chi-square or Fisher’s exact test, as appropriate according to the variables distribution, was used to compare categorical variables. Given the exploratory nature of this study of phase I, no multiplicity adjustments were considered. The analysis was performed using SPSS(^®^) v. 15 (SPSS Inc., Chicago, IL, USA) and the level of significance was established at the 0.05 level (two-sided).

## 3 Results

### 3.1 Participant flow and recruitment

The CT lasted 5 months (from 31th of March to 29^th^ of August 2014) from the data the first participant enrolled to the time the last participant finished. After screening a total of 76 volunteers during the whole CT, 51 were included and randomized. A total of 25 volunteers were excluded for not fulfilling the study’s requirements: presenting hematologic abnormalities such as anaemia (3 participants) or thrombocytopenia (2), vaccinated 3 months before the CT (2), and having an autoimmune disease (1). Recent vaccination and autoimmune disease were excluded as considered able to interfere with the immunological results. After groups with negative TST were completed, 14 screened participants had a TST negative result, and thus were not included. And finally, 3 participants were excluded because the recruitment period was promptly ended (Fig 1).

The other 51 volunteers were included and randomized. Eighteen participants were in the placebo group, 17 in the high dose NR and 16 in the low dose NR group.

Each volunteer was treated with the allocated intervention (NR or placebo) and followed-up for a total of 6 weeks. No loss of follow-up or discontinuation of the treatment occurred, with the all 51/51 volunteers being analysed at the end of the CT. None of the subjects included in the present CT decided to withdraw from the study, nor was removed by the investigators. All volunteers declared to take the whole treatment (14 vials on a daily basis).

All the results and data obtained from the volunteers during the whole study are considered confidential. Furthermore, the investigator’s team certifies that to their knowledge all subjects were healthy and thus considered able to be included in the CT, in terms of fulfilling all the study’s requirements.

The only protocol deviation from the study protocol occurred was the recruitment promptly stopped because the TST reagent (PPD RT-23) was out of stock (http://www.aemps.gob.es/informa/notasInformativas/medicamentosUsoHumano/problemasSuministro/2014/NI-MUH_20-2014-tuberculina.htm), resulting in a smaller sample size than expected (n = 60) in the TST-positive groups.

### 3.2 Baseline data

All demographic and clinical baseline characteristics for the 51 volunteers finally included in the CT, with the corresponding ratio or median with the interquartile range (IQR), are summarized in Table 2.

**Table 2:** Demographic characteristics of the clinical trial.

	Placebo (n = 18)	Nyaditum resae^®^ low dose (n = 16)	Nyaditum resae^®^ high dose (n = 17)
Age (years)*	25.5 (22–36.3)	31 (23–44.3)	29 (21.5–42.5)
**Gender (% men)**	28%	43.80%	52.90%
**Underlying disease (% yes)**	88.90%	93.80%	94.10%
Height (cm)*	165.5 (160.5–170.3)	169.0 (162.5–175.3)	171.0 (163.5–179.5)
Weight (kg)*	65.7 (51.8–79.9)	70.1 (58.8–80.8)	67.4 (61.5–81.5)
Systolic blood pressure (mm Hg)*	111 (102–122)	116 (103–141)	113 (106–123)
Diastolic blood pressure (mm Hg)*	64 (58–67)	65 (58–77)	63 (59–69)
Cardiac frequency*	69 (60–79)	64 (57–72)	68 (60–75)
Respiratory frequency*	18 (16–20)	20 (17–20)	18 (13–21)

* median (IQR)

The anamnesis and physical exams performed to the volunteers during screening did not show any clinical significant abnormality. The results of the laboratory analysis of all the volunteers included in the study performed during the screening were in the normal range; otherwise, the investigators considered them without clinical significance.

### 3.3 Outcomes and estimation

#### 3.3.1 Results of laboratory tests

The abnormal laboratory tests possibly or probably related to the investigational treatment recorded during the CT include kidney function, hepatic enzymes, haematology and glycemia. No significant statistical differences were found when compared between groups (Table 3).

**Table 3:** Number of subjects presenting possible or probable related adverse events.

ADVERSE EVENTS			Participants		Placebo (n = 18)	Nyaditum resae^®^ low dose (n = 16)	Nyaditum resae^®^ high dose (n = 17)	P-value
			N	%				
**Gastrointestinal**	Abdominal pain		18	35.3	8	3	7	0.221
	Stool frequency	Increased	18	35.3	8	6	4	0.412
	Stool consistency	Decreased	14	27.5	5	5	4	0.883
	Nausea		9	17.6	6	2	1	0.084
	Diarrhoea		6	11.8	3	2	1	0.588
	Dyspepsia		4	7.8	1	3	0	0.087
	Stool frequency	Decreased	3	5.9	3	0	0	0.054
	Stool consistency	Increased	3	5.9	3	0	0	0.054
	Vomits		2	3.9	1	0	1	0.462
	Flatulence		2	3.9	1	1	0	0.434
	Constipation		1	2	1	0	0	0.346
	Epigastralgia		1	2	1	0	0	0.346
	Rectal Tenesmus		1	2	0	1	0	0.307
**Non-Gastrointestinal**	Hepatic enzymes alterations		7	13.7	1	4	2	0.206
	Hematologic alterations		5	9.8	3	1	1	0.378
	Cephalea, migraine		4	7.9	1	3	0	0.346
	Respiratory Infection		3	5.9	1	1	1	0.996
	Hyperglycemia		2	3.9	0	1	1	0.221
	Hypoglycemia		1	2	0	0	1	0.346
	Thrombocytopenia		1	2	0	1	0	0.307
	Others		5	9.8	1	3	1	0.378

Seven participants (13.7%) presented an increase of bilirubin or transaminase values. All of the elevations were asymptomatic, mild and resolved in the following weeks or at the end of the trial.

Five participants (9.8%) presented a decrease of haemoglobin or an abnormal value of leucocytes, all of them were also asymptomatic, mild and resolved in the following weeks or at the end of the trial (excepting one volunteer in the placebo group with a probably myeloproliferative syndrome currently in study). A participant presented a moderate thrombocytopenia that spontaneously resolved within 5 weeks.

Hyperglycemia was recorded in 2 participants and hypoglycemia in one. These were also asymptomatic, mild and resolved in the following weeks or at the end of the trial.

#### 3.3.2 Physical examination and vital signs

No anomalies regarding to vital signs were detected during the trial visits.

#### 3.3.3 Adverse events

The 51 participants reported a total of 322 AE. The 92.5% (298) of the AE were mild, and only one was classified as severe but was considered improbably to be related to the investigational treatment (radial bone fracture after slipping and falling). No statistical differences were found when comparing the median (IQR) number of AE between the placebo group and both treatment groups (Table 4).

**Table 4:** Comparison of the median (IQR) number of AE by groups.

	Gastrointestinal	Non-Gastrointestinal	Total
**Placebo**	4 (2–6)	2 (1–3)	6 (4–9)
**Nyaditum resae**^**®**^ **low dose**	2.5 (1.3–4)	2 (1.3–4.5)	4.5 (4–9)
**Nyaditum resae**^**®**^ **high dose**	2 (1.0–3.5)	2 (1–4.5)	5 (2.5–7.5)
**P-value**	0.057	0.444	0.467

The 46.3% (149/322) of the overall reported AE were considered possibly or probably related to the investigational treatment. No AE were causally related as definite. The 47% of the AE probably or possibly related to the treatment (70/149) occurred in the placebo group, 29.5% (44/149) in the NR low dose group, and 23.5% (35/149) in the NR high dose group. None of them were severe (94% were mild and 6% moderate). The most common reported AE were gastrointestinal events (82%, 122/149).

The 78.4% (40/51) of the participants presented at least one gastrointestinal event (no significant differences were found between treatment groups, *p* = 0.078), reporting most frequently mild abdominal pain and/or increase of the stool frequency (in 35.3% of the participants each one [18/51]) (Table 3).

#### 3.3.4 Imunogenicity assays

According to the work of Dwyer [26] differential expression of CD25 and CD39 on circulating CD4+ T cells distinguishes between memory Treg (CD25+CD39+) and pathogenic cellular populations that secrete proinflammatory cytokines such as IL-17 (CD25-CD39+) and that an increase in the latter population is related with a decrease on the Treg population. Our data in the TB murine model of TB demonstrate that the evolution from LTBI to TB is also related to an increase of this CD25-CD39+ T cell population through the production of IL-17 [22]. FACS analysis strategy used allowed us to identify 4 different types of cells. **CD25+CD39-**: mainly effector Tregs (80–90%); **CD25+CD39+**: memory Tregs; **CD25-CD39-**: Non Treg Effector cells; **CD25-CD39+**: ex-Treg cells mainly IL-17 producers.

**Fig 3:**
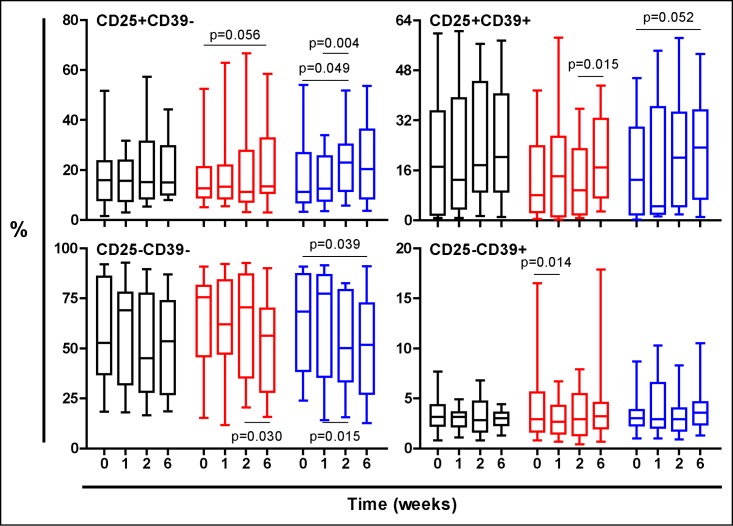
Evolution of the PPD-stimulated T cells in all volunteers regardless their TST status. Treatment groups are represented in black, red and blue, corresponding to Placebo, low dose and high dose Nyaditum resae^®^ respectively. P- values calculated by Wilcoxon matched pairs test. Plots are shown with median, IQR and minimum/maximum values.

**Fig 4:**
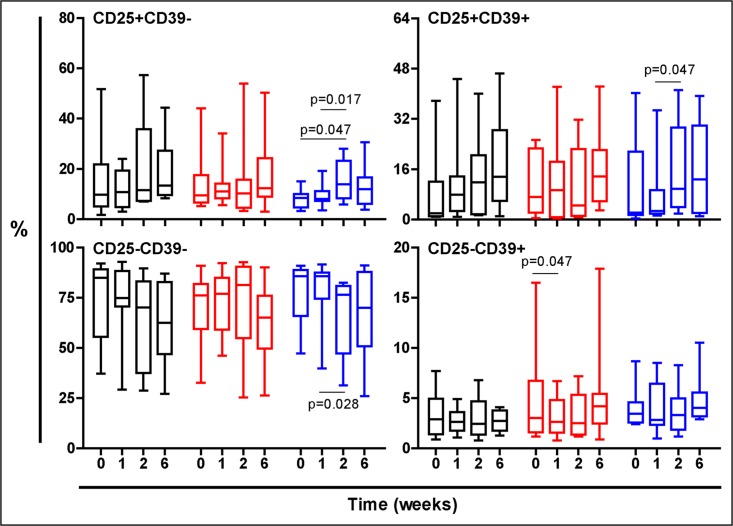
Evolution of the PPD-stimulated T cells in TST-negative volunteers. Treatment groups are represented in black, red and blue, corresponding to Placebo, low dose and high dose Nyaditum resae^®^ respectively. P- values calculated by Wilcoxon matched pairs test. Plots are shown with median, IQR and minimum/maximum values.

**Fig 5:**
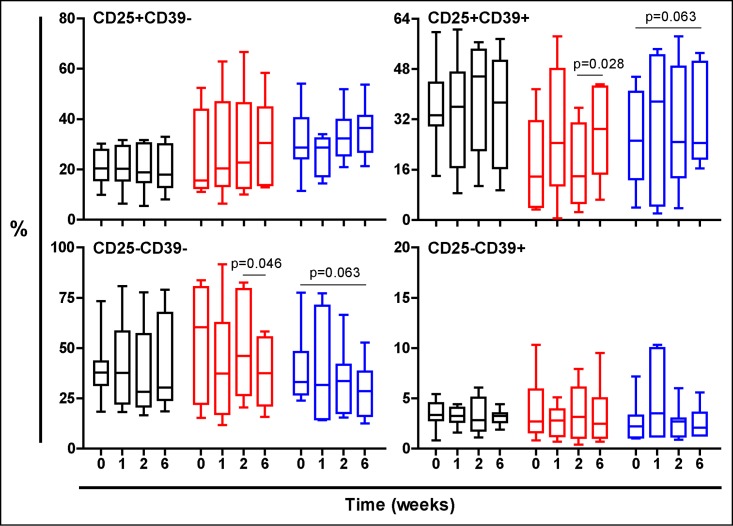
Evolution of the PPD-stimulated T cells in TST-positive volunteers. Treatment groups are represented in black, red and blue, corresponding to Placebo, low dose and high dose Nyaditum resae^®^ respectively. P- values calculated by Wilcoxon matched pairs test. Plots are shown with median, IQR and minimum/maximum values.

Figs 3, 4 and 5 show the evolution of the PPD stimulated cells. Essentially both TST-negative and TST-positive volunteers treated with NR experimented a global increase on Treg response, showed in both populations of CD25+CD39-, mainly effector Treg cells or CD25+CD39+ memory Treg cells, according to different authors [26,29].

**Fig 6:**
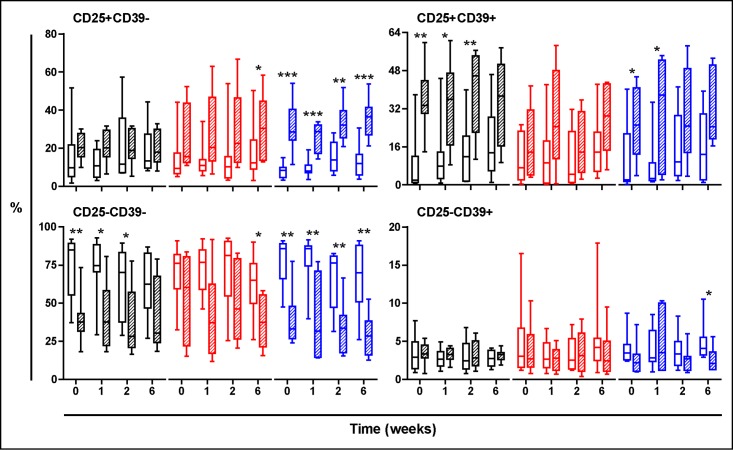
Evolution of the PPD-stimulated T cells according to TST status. Comparison between TST-negative and TST-positive volunteers. Treatment groups are represented in black, red and blue, corresponding to Placebo, low dose and high dose Nyaditum resae^®^ respectively. P- values calculated by Mann Whitney test, expressed in intervals: * from 0.05 to 0.01; ** from <0.01 to 0.001 and *** <0.001. Plots are shown with median, IQR and minimum/maximum values.

Furthermore, the levels of Tregs were in general higher in TST-positive subjects during and after the treatment, being the group treated with the high dose NR the one that experienced more significant increases with time (Fig 6). Indeed, in the group of TST-positive subjects treated with placebo the baseline levels were really high and stable, probably reflecting an already protected population. In some cases a significant decrease in the CD25-CD39+ population has also been noted.

**Fig 7:**
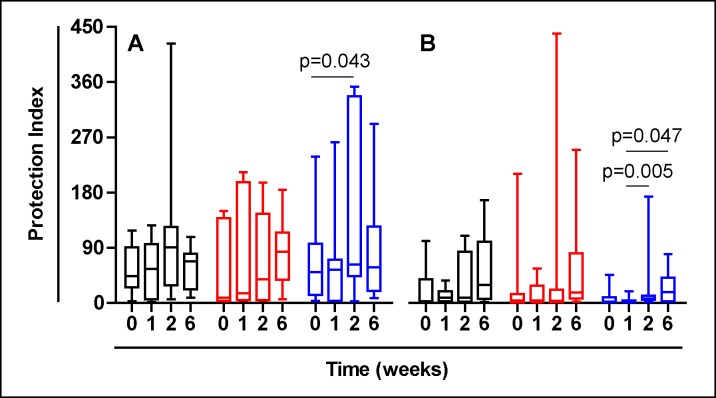
Protection index as assayed after Dwyer [26] determined as a ratio between the data obtained by the ratio of stimulated and non-stimulated CD25+CD39+ and the obtained by the ratio of stimulated and non-stimulated CD25-CD39+ cells. Results divided according TST status: (A) TST-positive, (B) TST-negative. Treatment groups are represented in black, red and blue, corresponding to Placebo, low dose and high dose Nyaditum resae^®^ respectively. P- values calculated by Wilcoxon matched pairs test. Plots are shown with median, IQR and minimum/maximum values.

Protection index as assayed by Dwyer [26] was also determined as a ratio between the data obtained by the ratio of stimulated and non-stimulated CD25+CD39+ and CD25-CD39+ cells. In this case, even when naturally Placebo groups can have increased levels of Tregs, only NR treated groups experienced an increase in this index, maybe relating to a “real” protection, or at least a better cellular distribution able to avoid a pro-inflammatory milieu. The levels were higher in TST-positive volunteers and after being treated with the highest dose of NR (Fig 7).

## 4 Discussion

The objectives of this CT were to demonstrate the safety of the new product Nyaditum resae^®^ and evaluate its effect on specific Regulatory T cells (Treg) cells population.

Surprisingly, the median number of AE in placebo treated participants was higher than in groups treated with the investigational treatment, but no significant differences were found when comparing between groups. More than two-thirds of participants presented a gastrointestinal event with a similar distribution between groups. Gastrointestinal adverse events are very unspecific and usually frequent when the investigational treatment is administered orally. Moreover, gastrointestinal events were those we could mainly expect because of administration route (oral) as well as the bacterial origin of the probiotic. As all the participants were informed about this potential effect, this could explain the high rate of AE considered probably and possibly. On the other hand, it is well known that the administration of placebo can induce AE, related to the informed potential AE of the investigational product. It is logical then the fact that placebo side effect profile is mostly similar to the side effect profile of the investigational treatment [30]. Variability on the adverse events profile in healthy volunteers has been related to personality and lifestyle of volunteers. Although randomisation, women were more frequent in the placebo group than in the investigational treatment groups. Somatization and hypocondriacal features, and adverse reaction in general, are predominantly related to women [31], which could explain the highest incidence in this group.

Laboratory abnormalities such as liver or glycemia alterations were more frequent in the probiotic groups than placebo, without significant differences found. Contrary to these alterations, leucocytes abnormalities were more frequent in the placebo groups, also without significant differences. The small sample size of this study is a limitation and could difficult the possibility to find statistical significant differences between groups. A bigger sample size could be necessary to elucidate the incidence and relation to treatment of those rare AE.

In the present clinical trial we have demonstrated the safety of the NR, because, although the AE were frequent, they were mild and spontaneously resolved within the following weeks, and no significant differences were found when compared between groups.

Data provided show for the first time that the administration of low doses of heat-killed non tuberculous mycobacteria is able to induce both effector and memory specific Tregs. The induction of Tregs by probiotics is well known [32] even when using heat-killed probiotics [33]. It has been recently demonstrated that in some cases the effect induced by probiotics is not affected by its viability [34], emerging the concept of “paraprobiotic” or “ghost probiotics” [35] which have clear advantages in terms of production, stability and security of the product. Shinkai *et al* [36] have recently evaluated the usefulness of the oral intake of heat-killed *Lactobacillus pentosus* strain b240 as immunoprotective by reducing the incidence rate of the common cold in elderly adults. Previously, Zhang *et al* [37] demonstrated that the administration of heat-killed *Enterococcus faecalis* FK-23 was able to attenuate the Th17 response in the lung, thus suppressing the allergic response in a murine model of asthma induced by ovalbumin.

Recently it has been demonstrated that IL-17 plays a paramount role in the evolution from infection to disease in a TB model in mice, by increasing the inflammatory response in the granuloma through a neutrophilic infiltration, followed by the coalescence of different lesions [8,9,22]. Furthermore, it has been demonstrated that mice from a strain that never develops TB had higher levels of Tregs and when these mice were treated with mAbs anti CD25 for the Tregs depletion, TB appeared [22].

After the induction of a low dose tolerance with a daily oral administration of heat killed mycobacteria, progression towards TB was abrogated as demonstrated by increasing from 30 to 50% the survival time of the mice. When translating this phenomenon to humans, another local defence mechanism not present in mice must be taken into account: the septae that surrounds secondary lung lobules able to encapsulate lesions and abrogate its growth [38].

So far, the induction of Tregs after Mtb infection has been understood as deleterious. Tregs have been claimed to be responsible for a weaker cellular immune response, stopping Th1 cellular proliferation [29] that would fuel the progression from LTBI to TB [39,40], and inducing a limited protection after BCG vaccination [41]. Further investigations have given them a neutral role [42–44] mostly in models where protection was measured through the reduction of the bacillary load. Recently, when also evaluating the role of the pathology, some authors have seen a protective role of the presence of Tregs linked to a control of the inflammatory response [45].

The presence of *M*. *fortuitum* complex bacilli in the tap water has been demonstrated by different authors in different regions of the planet [46–51] including the region where the clinical trial was run [52]. This was the reason for developing NR as a supplement food once an equivalent protective effect than the induced by the HKMtb was demonstrated [22]. On the other hand this fact might had played a role in our trial as there was a risk that subjects from the placebo group had drank tap water with *M*. *fortuitum* bacilli, or even that treatment with the investigation product boosted the immune response of subjects that has been previously in contact with this bacillus. When designing the trial, we assumed to include this risk, as it is part of real life.

NR has also demonstrated its capacity to induce specific Tregs and also memory Tregs (CD39+). Overall, the highest dose was more clearly related to an increase of this cell population. TST status has also shown to play a major role. TST positive participants had in general more Tregs than TST-negative, both in Placebo or NR treated subjects. This fact was expected because Mtb infection itself also induces the presence of Tregs. Interestingly, the Placebo group of TST-positive subjects had a very high number of Tregs from the baseline, although it did not experiment any increase during the clinical trial. In this regard we could theorize that those subjects have already been protected, and it can be hypothesized that this is either because they have had contact with non-tuberculosis mycobacteria or because the Mtb infection has naturally triggered a higher proportion of Tregs in those volunteers. This latter hypothesis would be in concordance with the concept of genetic resistance [53,54]. In fact, in our experience, we have been able to demonstrate higher Treg % in those mice strains that better resist the Mtb infection (data not published).

Regarding the data presented to monitor Treg response, we mainly presented raw data of PPD stimulated PBMCs, following the methodology of other authors [28] that have observed a better response-window. In fact, non-stimulated PBMCs have shown very low levels of Tregs through time in all treatment groups. When we analysed the protection ratio as a measure of protection firstly described by Dwyer et al [26], we have been able to show a significant increase through time in the group of the highest dose of NR (10^5^ CFU) in both TST-positive and negative participants.

In conclusion, data supports that the administration of Nyaditum resae^®^, a product based on heat killed bacilli of *Mycobacterium manresensis*, is able to induce a specific increase of the Treg response including memory cells, with an excellent safety profile, thus being a new tool to reduce the risk of the progression from latent infection to TB in humans.

## Supporting information

S1 File Consort checklist. (PDF)

S2 File Nyadatreg protocol (English version). (PDF)

S3 File Annex 1 & 2: Data collection notebook & SAE form. (PDF)

S4 File Annex 4: Technical file of the product. (PDF)

S5 File Annex 5: Participant information sheet. (PDF)

S6 File Annex 6: Informed consent for participants. (PDF)

S7 File Annex 7: Patient diary. (PDF)

S8 File Annex 8: Inclusion diagram. (PDF)
