# Supplementary material for: Pilot, double-blind, randomized, placebo-controlled clinical trial of the supplement food Nyaditum resae® in adults with or without latent TB infection: Safety and immunogenicity
Source: PLoS One. 2017 Feb 9;12(2):e0171294. doi: 10.1371/journal.pone.0171294 (PMC5300153; doi:10.1371/journal.pone.0171294)
Supplement: S2 File — (PDF) [file pone.0171294.s002.pdf]

Double-blind, randomized, placebo-controlled, masked, clinical trial to evaluate the tolerability and immunogenicity of the probiotic Nyaditum resae® administered to adults with or without latent tuberculosis infection.

Code: NYADATREG

Final Version, 3rd February 2014

Sponsor:

Manresana de Micobacteriologia, SL.
C/ Urgell n°26, 1
08240-Manresa

Principal Investigator:

Dr. Eva Montané Esteva

PRINCIPAL INVESTIGATOR AND SPONSOR SIGNATURES

The researchers responsible for the clinical trial: **Double-blind, randomized, placebo-controlled, masked, clinical trial to evaluate the tolerability and immunogenicity of the probiotic Nyaditum resae® administered to adults with or without latent tuberculosis infection.**

Declare that the clinical trial will be conducted following the protocol.
Changes in protocol will be presented with the agreement of the researchers and the sponsor.

Principal Investigator: Dr. Eva Montané Esteva

Signature and date

Promotor: Dr. Pere Joan Cardona
Manresana de Micobacteriologia, SL

Signature and date

SUMMARY

Identification and address of the sponsor
Manresana de Micobacteriologia, SL
C/ Urgell nº 26,1
08240-Manresa

Study title
Double-blind, randomized, placebo-controlled, masked, clinical trial to evaluate the tolerability and immunogenicity of the probiotic Nyaditum resae® administered to adults with or without latent tuberculosis infection.

Protocol code
NYADATREG

Principal investigators and address
Dra. Ana Mª Barriocanal Barriocanal (abarrio.germanstrias@gencat.cat)
Dra. Eva Montané Esteva (emontane.germanstrias@gencat.cat)
Servei de Farmacologia Clínica
Hospital Universitari Germans Trias i Pujol
Ctra. de Canyet, s/n
08916 Badalona
Tel. 93 497 88 65 / 93 497 84 88

Kind of centre where the study is planned to be carried out
German Trias i Pujol University Hospital

Research Ethics Committee
CEIC Hospital Universitari Germans Trias i Pujol
Hospital Universitari Germans Trias i Pujol
Carretera de Canyet s/n
08916 Badalona (Barcelona)

Main objective
Evaluate the tolerability and immunogenicity of 2 doses of Nyaditum resae®

Design
Double-blind, three-armed parallel, randomized, placebo-controlled and masked single-centre clinical trial

Disease under study
Tuberculosis

Information about the probiotic under study
Nyaditum resae® contains bacilli of *Mycobacterium fortuitum* strain "Manresa" heat-killed, lyophilized and encapsulated.

Population under study and total number of participants
Adults with or without latent tuberculosis infection. A total of 60 people.

Calendar
Presentation to research ethics committee: November, 2013.
Beginning of trial (inclusion of the first participant): January, 2014.
Inclusion period: January-April, 2014.
Final report: August-September, 2014.

Funding source
Manresana de Micobacteriologia SL is in charge of the cost of the study.

TABLE OF CONTENTS

1. SUMMARY ... 3
1.1 Identification and address of the sponsor ... 3
1.2 Study title ... 3
1.3 Protocol code ... 3
1.4 Principal investigators and address ... 3
1.5 Kind of centre where the study is planned to be carried out ... 3
1.6 Research Ethics Committee ... 3
1.7 Main objective ... 3
1.8 Design ... 3
1.9 Disease under study ... 3
1.10 Information about the probiotic under study ... 3
1.11 Population under study and total number of participants ... 3
1.12 Calendar ... 3
1.13 Funding source ... 3
2. TABLE OF CONTENTS ... 4
3. GENERAL INFORMATION ... 5
3.1 Code ... 5
3.2 Title ... 5
3.3 Information about the sponsor and the CRO ... 5
3.4 Information about the principal investigator and collaborators ... 5
3.5 Center where the study is planned to be carried out ... 5
3.6 Involved services ... 5
3.7 Expected duration ... 5
4. JUSTIFICATION OF THE STUDY: CRITICAL REVIEW OF LITERATURE ... 6
4.1 Introduction ... 6
4.2 Tuberculosis is an inflammatory disease ... 7

GENERAL INFORMATION

Code

NYADATREG

Title

Double-blind, randomized, placebo-controlled, masked, clinical trial to evaluate the tolerability and immunogenicity of the probiotic Nyaditum resae® administered to adults with or without latent tuberculosis infection.

Information about the sponsor and the CRO

Sponsor:
Manresana de Micobacteriologia, SL
C/ Urgell nº 26,1
08240 Manresa
CRO:
FLS-Research Support
Hospital Universitari Germans Trias i Pujol
Ctra. de Canyet, s/n
08916 Badalona
Tel. 93 497 84 14
Fax. 93 465 76 02
rescrig@fls-rs.com

Information about the principal investigator and collaborators

Dra. Eva Montané Esteva (emontane.germanstrias@gencat.cat)
Servei de Farmacologia Clínica
Hospital Universitari Germans Trias i Pujol
Ctra. de Canyet, s/n
08916 Badalona
Tel. 93 497 88 65
Colaborators:
Dr. Ana Mª Barriocanal Barriocanal
Dr. Ana Lucía Arellano Andrino

Center where the study is planned to be carried out

Hospital Universitari Germans Trias i Pujol (HUGTiP)
Carretera de Canyet s/n
08916 Badalona (Barcelona)

Involved services

- Patients follow-up: Clinical Pharmacology Service
- Analytic determinations: Biochemistry, Hematology and Microbiology Services from HUGTiP
- Immunologic determinations: Unitat de Tuberculosis Experimental, Fundació Institut Germans Trias i Pujol.
- Imaging tests: Radiology Service from HUGTiP

Expected duration

The duration of this study from the wording of the protocol until the final report is about 1 year (from November 2013 to September 2014). Here bellow is the estimated schedule:

- Presentation to research ethics committee: November, 2013.
- Beginning of trial (inclusion of the first volunteer): January, 2014.
- Enrolment period: January-April, 2014.
- Follow-up date of the last enrolled participant: June, 2014
- Final report: August-September, 2014.

JUSTIFICATION OF THE STUDY: CRITICAL REVIEW OF LITERATURE

Introduction

The incidence of tuberculosis is still a problem of first magnitude. Every year 1.5 million people die worldwide; there are 10 million cases of illness and 100 million new infected. The growing problem of multi-resistance should also be considered: around 700,000 patients, a figure that increases annually by 100,000 people (1).

It is estimated that one third of the world population suffers from latent tuberculosis infection (LTBI). Only 5% of those infected people develop the disease in the following 10 years from the moment of the beginning of the infection; the other 95% remain with LTBI (Figures 1 and 2), which goes unnoticed unless they have specific diagnostic tests performed (2).

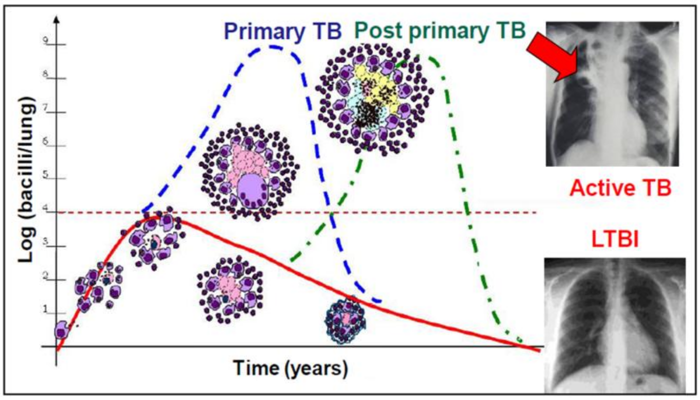

Figure 1. Evolution of TB infection in the human hostage. Once the specific immune response towards the tuberculosis bacillus has been originated, the infection can evolve to illness and cause primary TB or become LTBI, which can lead towards post-primary tuberculosis. The probability of developing the disease decreases with time.

The prevention of TB is currently very difficult, it is an infection caused by *Mycobacterium tuberculosis*, which is transmitted by air. There is no well-identified risk factor for becoming infected and there is still no prophylactic vaccine to prevent the infection. Since 15 years ago many efforts have been devoted to finding a vaccine, and in fact there are many candidates (3, 4). All of these vaccines have in common the search for a powerful antigen, able to generate a Th1 response that can generate interferon gamma to activate infected macrophages and destroy the bacilli inside (3). Unfortunately, although the reduction in bacterial population has been achieved in experimental models, it still hasn't been validated that it is this process indeed the one that protects against infection and disease (5). On the other hand, this is precisely the mechanism that generates BCG, Bacillus Calmette Guerin, attenuated strain of *Mycobacterium bovis*, which is administered from 1927 in neonates. In this case, unfortunately, it has been proven that it is not able to avoid infection, but it provides protection against the acquisition of a deadly disease (meningeal or miliary tuberculosis) (6). The problem is that despite having the best of immunities, once the bacilli has entered the pulmonary alveoli, has been phagocytized by an alveolar macrophage and has begun to grow, the infection, and in most cases reinfection, has been already set. In fact, this is the origin of the Dynamic Hypothesis through which our group explains the maintenance of latent infection (LTBI) (7).

However, the most crucial issue in tuberculosis is the induction of the disease, that is the passage of LTBI to active tuberculosis (Figure 2), and so far there is no theory that can explain this phenomenon in a convenient way.

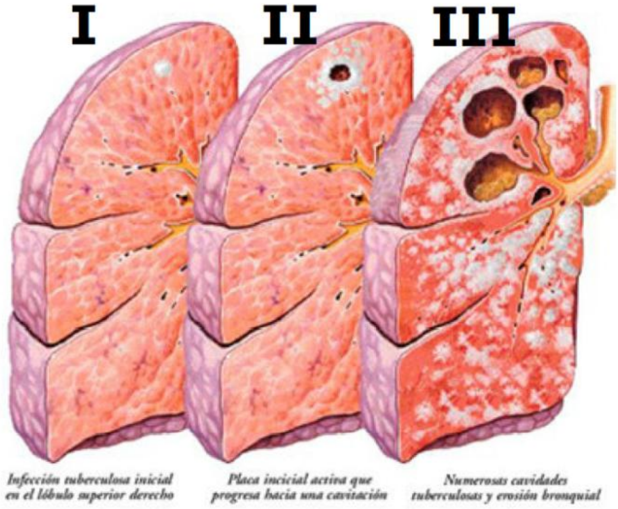

Figure 2. This diagram illustrates the development of the disease in humans. In most cases the lesion

caused by *Mycobacterium tuberculosis* does not progress from a small lesion (I), while in a small percentage (10%) of infected people, the lesion may progress to cavity, generating disease (II and III).
(Extracted from Netterimages.com)

Tuberculosis is an inflammatory disease

In this sense, liquefaction, i.e. the conversion of necrotic tissue into a liquid product, is considered key to the development of the disease. For this reason, since 2009, in the Experimental Tuberculosis Unit we have focused on the search of a liquefaction model in mice. Previous experiences (8) led us to discover the induction of this liquefaction in an immunosuppressed mice experimental model (SCID) after antibiotic treatment and leaving to reactivate the bacillary growth in old lesions.
Meanwhile, working in the pig model, studying this process in large mammal, we realized that the lung tissue of big mammals had very thin tendons that allow maintaining the lung's structure and are sensitive to any mechanical change in the lung. These tendons were so sensitive, that they were able to detect lesions from *Mycobacterium tuberculosis* infection (0.5 mm) and encapsulate them within a week (9). In this context, the development of the disease was an evident problem (Figure 3).

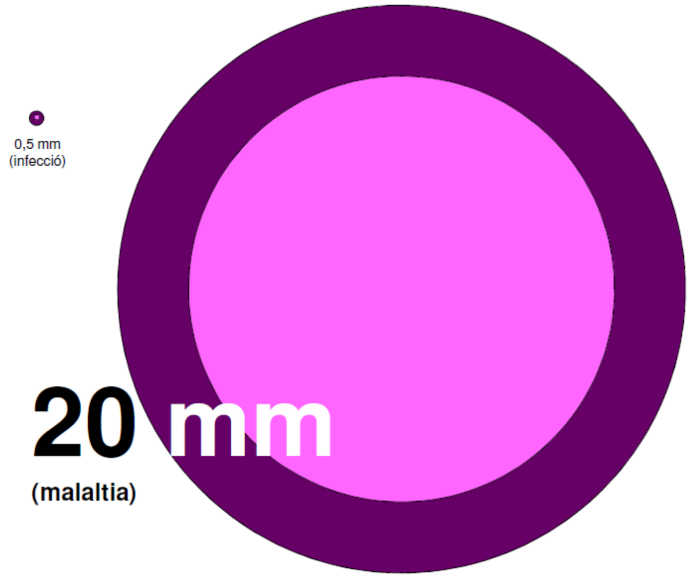

Figure 3. In the pigs experimental model, we realized that the problem of the disease development had an important mechanical component. The infection lesions were really small (0.5 mm) and tended to be quickly encapsulated within a week. In humans, it is empirically known that to generate a cavity a lesion of at least 20 mm is needed. How is this problem possible? *(objects measures proportional to real measures)*

Working with a model of described a few years ago, the "Kramnik model" (10), we confirmed that there was at least one type of mouse that naturally generated liquefaction lesions. This fact caused them an immediate death. Studying this model we found the answer to the problem. Lesions grew faster individually than in most mice models due to the accumulation of neutrophils. Later, if there were several lesions together, they would converge making it a very big lesion within 12 days (Figure 4).

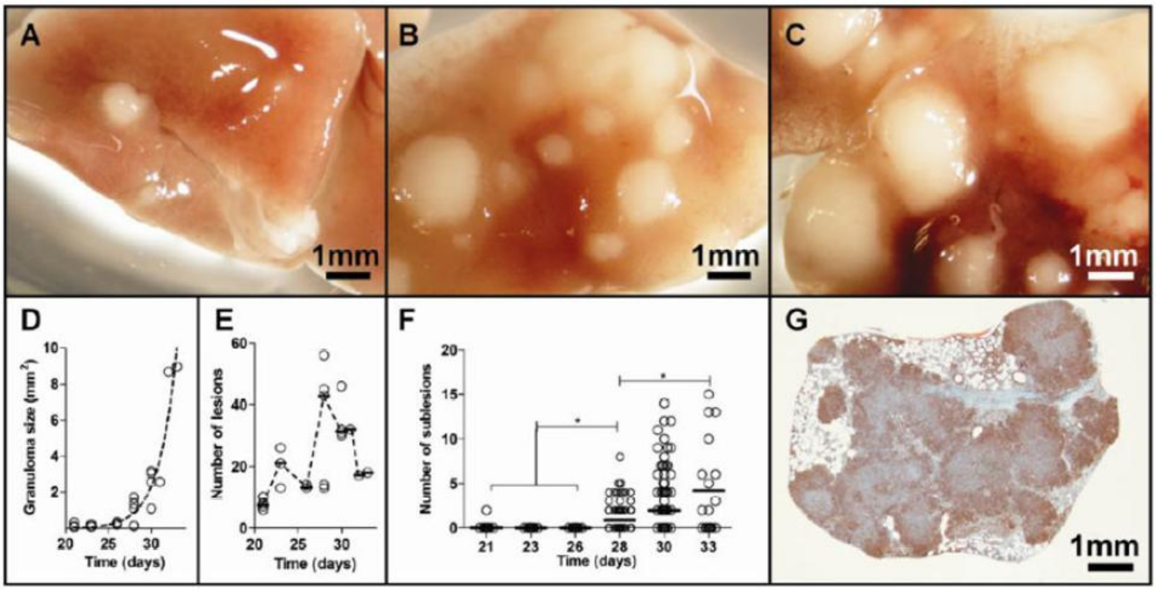

Figure 4. Progression of lesions in the Kramnik experimental model, in which lesions grow individually to later converge into a bigger one (11) (*Marzo et al; Tuberculosis 2013*), solving the posed problem with the pig model.

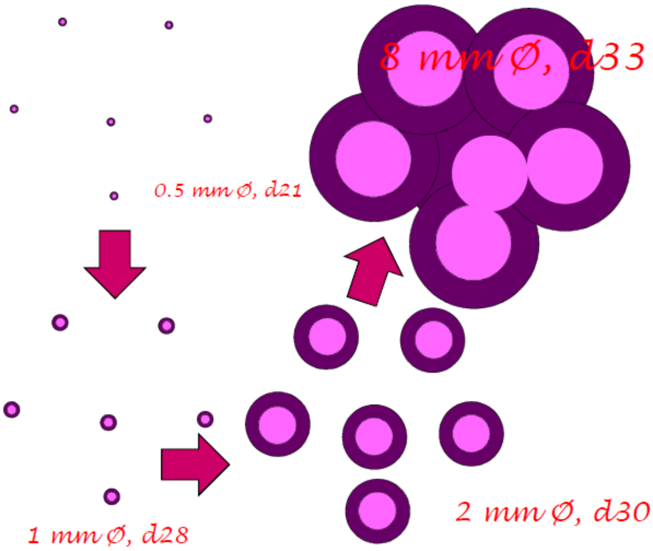

Figure 5. Interpretation diagram of the results obtained in the mouse experimental model of tuberculosis (Kramnik model)

Thus, the induction of tuberculosis have an important inflammatory component. This fact was confirmed once it was demonstrated that the administration of low dose aspirin or ibuprofen diminished or avoided mortality in animals significantly *(11)*.

4.3. Oral tolerance: the origin of Nyaditum resae®

In this regard, within the Experimental Tuberculosis Unit, took it a step further, as the continuous administration of anti-inflammatories is an obvious risk to the health of patients. The move was a reaction to the induction of specific oral tolerance against tuberculosis bacillus. In terms of immune tolerance has been defined as "any mechanism by which it is prevention, suppression or potentially harmful immune response, or a response revert to non-harmful" (12). Oral tolerance is a well-known mechanism of induction of tolerance, thus preventing related hypersensitivity reactions against food proteins or antigens bacterial intestinal flora (12). Specifically, there are many experiences that demonstrate the continued administration of low

doses of antigen manage to reduce Th1 responses while maintaining the Th2 response and Th3 cells or Treg cells (12-14).

So we began our experiences managing our animals before or after infection by Mycobacterium tuberculosis, the bacillus doses of the same dead heat. And we managed to increase the survival of animals and reduce the inflammatory response, similar to how he had obtained after administration of ibuprofen or aspirin (Figure 6).

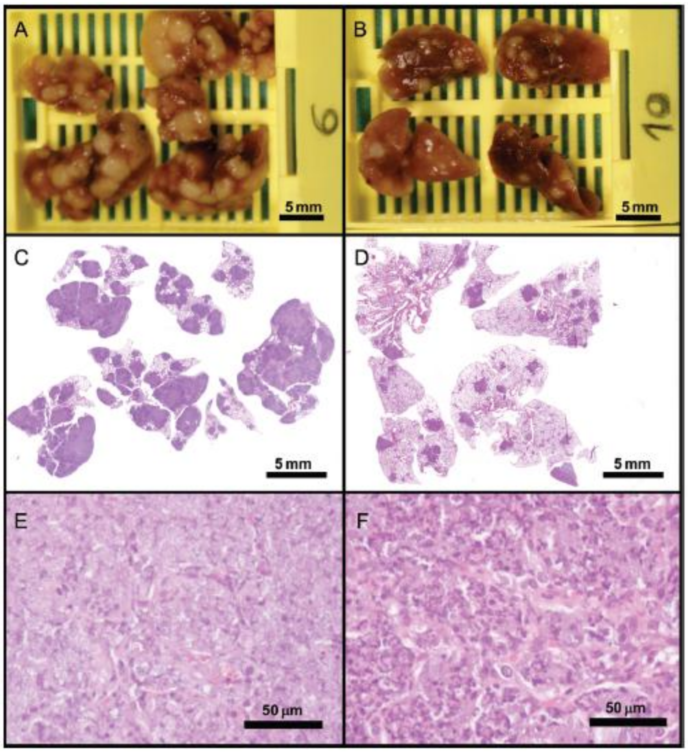

Figura 6:

Antiinflammatory effect achieved with administration Nyaditum resae® model of TB disease in mouse model (Kramnik) . The samples corresponding to 28 days post infection when the control animals that do not take the treatment , they begin to die (Figures A, C and E). While those who continued treatment do not (figure B , D and F).

We evolve this concept to make this technology more affordable . The idea was to get the same effect by using bacteria of the family of *Mycobacterium tuberculosis* , which are so common in the environment and have no pathogenic activity . In this regard we chose *Mycobacterium fortuitum* , because it is a common bacteria that normally colonize the water we drink and can be found in the intestinal flora . The Nyaditum resae® then manufactured from heat-killed bacilli (autoclavats) strain of *Mycobacterium fortuitum* Manresa.

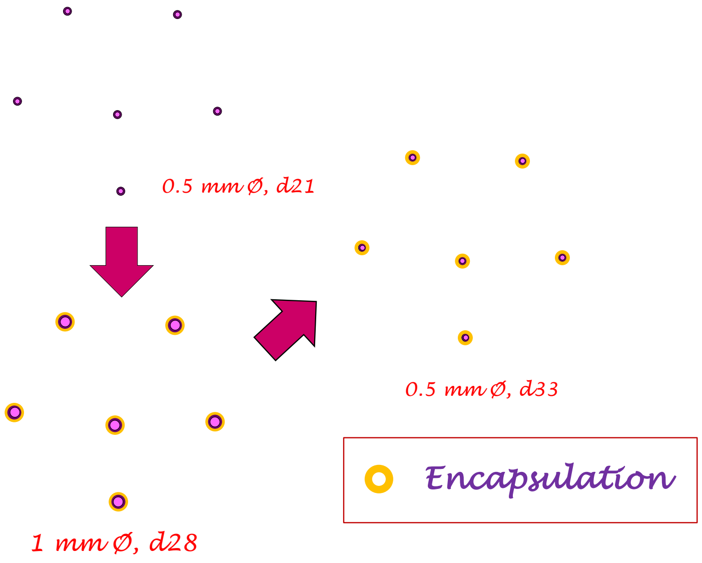

Figura 7:

Interpretation of the protective role of Nyaditum resae® . In the case of humans, the deficit would inflammatory response in the lung long enough to encapsulate injuries and prevent disease.

As indicated in Figure 7, the mechanism of action of Nyaditum resae® be based on maintaining an anti-inflammatory response, which in the lungs of humans would be sufficient to act encapsulation system injuries, preventing its growth individual and subsequent coalescence to build cavitation damage. How this is achieved tolerant inflammatory response? Thus causing the formation of regulatory T cells. And as illustrated in Figure 8 and schematically, the immune response is generated when Dendritic cells ("Mature DC" on the scheme) are antigens to generate effector T cells ("Effector cells") . In the case of TB, these effector cells are of two types: the Th1, which generates interferon-gamma, to activate macrophages infected and Th17, that attract neutrophils. Induction of regulatory cells (Treg) are antagonists and Th17 thus inducing Tregs blocks the inflammatory response in people that Th17 response is too important (15). In people that this answer is discreet and normal tregs are not attracted to this process and do not act because they are not needed.

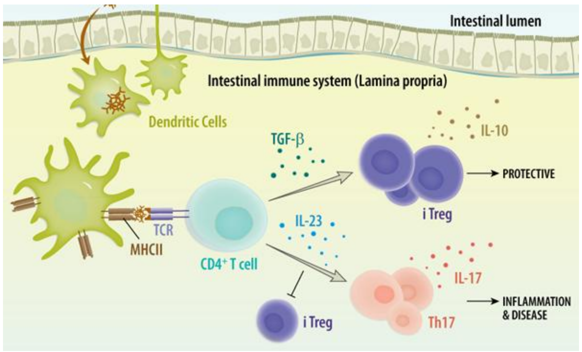

Figure 8:

Diagram of the immune response from publishing Walker LSK . (Immunology 2004) that shows the action of the immune system against tuberculosis .

After demonstrating the presence of these specific Treg against Mycobacterium tuberculosis in mice treated with Nyaditum resae® Kramnik in the model , we evaluated this marker in a co-inventor of researchers Nyaditum resae (PJC) , which offer to take the full treatment (14 capsules Nyaditum resae® 105 UFCs , one every 24 hours). Preliminary results are presented in Figure 9. After the 7th administration, detected a significant increase in Treg cells (CD4 + CD25 + CD39 +) specific BCG and PPD after 7 days of incubation , indicating the appearance of specificity and memory of these tregs . There were any adverse event .

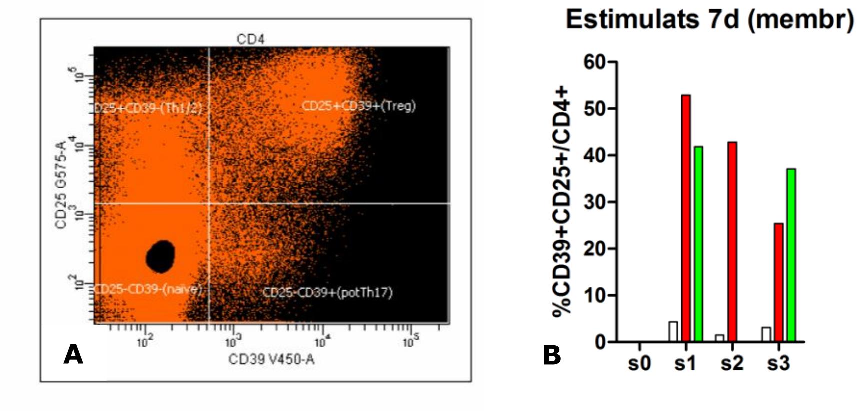

Figura 9:
Increased memory Treg cells in peripheral blood volunteer who has Nyaditum resae® administered for 14 days . Figure A shows the analysis in flow cytometry . Figure B results before (s0) and after 1, 2 and 3 weeks after starting treatment , where bars show the percentage of Tregs after PBMCs stimulated with PPD (red), BCG (green) stimulate or without (white) .

In fact, the presence of these stem cells has been significantly • in patients with active tuberculosis, and therefore have turned this defense mechanism to prevent excessive inflammatory response (16) . In fact , the increase of this population has been associated with a decrease in the levels of IL -17 in the serum of subjects infected with M. tuberculosis (17) . Finally, this type of Treg has been identified as a factor of good prognosis in patients transplanted in comparison to the population of CD4 + CD25 - CD39 + population represents Th17 , inflammatory , related to rejection of the organ (18) .

4.4. References

1. Eurosurveillance editorial t. ECDC and WHO/Europe joint report on tuberculosis surveillance and monitoring in Europe. Euro surveillance : bulletin Europeen sur les maladies transmissibles = European communicable disease bulletin. 2013;18(12).
2. Cardona P, Ruiz-Manzano J. On the nature of Mycobacterium tuberculosis-latent bacilli. The European respiratory journal. 2004;24(6):1044-51.
3. Brennan MJ, Thole J. Tuberculosis vaccines: a strategic blueprint for the next decade. Tuberculosis. 2012;92 Suppl 1:S6-13.
4. Ottenhoff TH, Kaufmann SH. Vaccines against tuberculosis: where are we and where do we need to go? PLoS pathogens. 2012;8(5):e1002607.
5. Ottenhoff TH, Ellner JJ, Kaufmann SH. Ten challenges for TB biomarkers. Tuberculosis. 2012;92 Suppl 1:S17-20.
6. Andersen P, Doherty T. The success and failure of BCG - implications for a novel tuberculosis vaccine. Nat Rev Microbiol. 2005;3(8):656-62.
7. Cardona PJ. A dynamic reinfection hypothesis of latent tuberculosis infection. Infection. 2009;37(2):80-6.
8. Guirado E, Amat I, Gil O, Díaz J, Arcos V, Caceres N, et al. Passive serum therapy with polyclonal antibodies against Mycobacterium tuberculosis protects against post-chemotherapy relapse of tuberculosis infection in SCID mice. Microbes and infection / Institut Pasteur. 2006;8(5):1252-9.
9. Gil O, Díaz I, Vilaplana C, Tapia G, Díaz J, Fort M, et al. Granuloma encapsulation is a key factor for containing tuberculosis infection in minipigs. PloS one. 2010;5(4):e10030.
10. Yan BS, Kirby A, Shebzukhov YV, Daly MJ, Kramnik I. Genetic architecture of tuberculosis resistance in a mouse model of infection. Genes and immunity. 2006;7(3):201-10.
11. Vilaplana C, Marzo E, Tapia G, Diaz J, Garcia V, Cardona PJ. Ibuprofen therapy resulted in significantly decreased tissue bacillary loads and increased survival in a new murine experimental model of active tuberculosis. The Journal of infectious diseases. 2013;208(2):199-202.

12. Weiner HL. Induction and mechanism of action of transforming growth factor-beta-secreting Th3 regulatory cells. Immunological reviews. 2001;182:207-14.
13. Yoshida T, Hachimura S, Kaminogawa S. The oral administration of low-dose antigen induces activation followed by tolerization, while high-dose antigen induces tolerance without activation. Clinical immunology and immunopathology. 1997;82(3):207-15.
14. Zhang X, Izikson L, Liu L, Weiner HL. Activation of CD25(+)CD4(+) regulatory T cells by oral antigen administration. Journal of immunology. 2001;167(8):4245-53.
15. Zhou L, Lopes JE, Chong MM, Ivanov, II, Min R, Victora GD, et al. TGF-beta-induced Foxp3 inhibits T(H)17 cell differentiation by antagonizing RORgammat function. Nature. 2008;453(7192):236-40.
16. Chiacchio T, Casetti R, Butera O, Vanini V, Carrara S, Girardi E, et al. Characterization of regulatory T cells identified as CD4(+)CD25(high)CD39(+) in patients with active tuberculosis. Clinical and experimental immunology. 2009;156(3):463-70.
17. de Cassan SC, Pathan AA, Sander CR, Minassian A, Rowland R, Hill AV, et al. Investigating the induction of vaccine-induced Th17 and regulatory T cells in healthy, Mycobacterium bovis BCG-immunized adults vaccinated with a new tuberculosis vaccine, MVA85A. Clinical and vaccine immunology : CVI. 2010;17(7):1066-73.
18. Dwyer KM, Hanidziar D, Putheti P, Hill PA, Pommey S, McRae JL, et al. Expression of CD39 by human peripheral blood CD4+ CD25+ T cells denotes a regulatory memory phenotype. American journal of transplantation : official journal of the American Society of Transplantation and the American Society of Transplant Surgeons. 2010;10(11):2410-20.

5. OBJECTIVES

5.1. Hipetesis

The administration of a low concentration (10^4 or 10^5 colony forming units - UFCs-) bacilli of Mycobacterium fortuitum strain Manresa dead heat for 14 days orally is well tolerated and produces a population of regulatory T cells • Cell specific memory

5.2. Objectives

1. Evaluation of the tolerability of two doses of probiotic Nyaditum resae® (CFUs 10^4 and 10^5), compared with placebo , in adults with or without latent tuberculosis infection (LTBI) .

2. Evaluation of immunogenicity with the production of Treg cells specific memory cells one week after the administration of Nyaditum resae® compared with placebo.

6. SOURCE OF INFORMATION AND SCOPE

The source document is the clinical history .

We design a notebook designed ad hoc data collection because the researcher fills in real time , ie , the information provides notebook data collection (QRD) shall be completed during the visits of the study.

Hospital , Multipurpose Unit of Clinical Research and Clinical Pharmacology .

7. STUDY DESIGN

Pilot clinical trial, double-blind, parallel • 3 branches parallel, randomized, placebo-controlled and masked.

7.1. Defining the study population:

Inclusion criteria:

1. informed consent before starting the selection procedures.

2. Women and men ≥ 18 years.

3. Willingness to fulfill the requirements of the protocol.

Exclusion criteria:

1. HIV positive.

2. known immunodeficiencies.

3. Pregnancy and lactation. *

4. active TB.

5. Participation in another clinical trial.

6. Chronic Administration: methotrexate, azathioprine, cyclophosphamide, oral corticosteroids and other immunosuppressive therapies / immunomodulatory.

7. Managing blood products or blood derivatives during the 6 months prior to randomization.

8. Detection by researcher lack of knowledge or will to participate and fulfill all the requirements of the protocol.

9. Any other finding that the investigator's opinion may compromise the performance of the protocol or significantly influence the results or interpretation of the effects of probiotic.

* The participants agree to use a contraceptive method duration of the study.

7.2. Criteria for withdrawal or abandonment

The subjects who have taken less than 5 doses of treatment, be excluded from the study. The subjects will end the trial before the time stipulated in the following circumstances :
• Loss Tracking
• Death
• Process or intercurrent disease that in the opinion of the investigator requires the removal of the subject.
• Deviation from the protocol in view of the developer requires the removal of the subject.
• The subject does not wish to continue with the trial.

7.2.1. Medical treatment before withdrawal

If there are any withdrawal must fill in the completion of study of QRD.

Be contributed detailed information about the date and reasons for termination promoter. That is, as a general rule, all subjects completed treatment prematurely will undergo a clinical examination and all tests specified in the last visit.

The investigator will provide medical support necessary to subject prematurely end the trial.

7.2.2. Losses pre-baseline visit

The subjects that do not meet the selection criteria after the visit of selection will take into account, but the record why the subject has not been included.

7.3. Observation period

The subjects will be followed for 6 weeks from the start of treatment.

7.4. Description and definition of treatment exposure

The subjects stratified according latent tuberculosis infection (LTBI, see definition section 8), separating positive for LTBI (LTBIpos) and negative LTBI (LTBIneg).

There will be three branches of treatment :

• Group A: Nyaditum resae® UFC 10^4

• Group B: Nyaditum resae® UFC 10^5

• Group C: Placebo

The placebo containing the same excipients that Nyaditum resae® .

Once randomized subjects will receive a total of 14 vials drinkable Nyaditum resae® or placebo. A vial administered orally daily in the morning with breakfast for 2 weeks . Participants will be followed for a total of six weeks after the start of treatment .

A total of 60 subjects will be randomized (30 LTBIpos LTBIneg 30) to one of six treatment groups , as presented in Table 1:

Group		# de subjects & LTBI status	Composition	Dose	Route
1	A1	10 LTBIpos	Nyaditum resae® 10^4	The subjects will receive 14 vials drinkable with Nyaditum resae® 10^4 UFCs	Oral
2	A2	10 LTBIneg			
3	B1	10 LTBIpos	Nyaditum resae® 10^5	The subjects will receive 14 vials drinkable with Nyaditum resae® 10^5 UFCs	Oral
4	B2	10 LTBIneg			
5	C1	10 LTBIpos	Placebo	The subjects will receive 14 vials drinkable with Placebo	Oral
6	C2	10 LTBIneg			

7.5. Randomization

Subjects randomly assigned at the time of inclusion in proportion 1: 1: 1 groups A, B and C through a randomization table. This table will be stratified according to whether or not the individual has latent TB to ensure a similar distribution in three branches. Table randomization will be done via a uniform distribution and assigning a range of values for each group and swinging every six subjects. The process of randomization will be done centrally from CRO FLS-Research Support. At the time of inclusion, researchers call the CRO indicating whether or not the subject has LTBI. The CRO will add to the list of subject randomization and will be assigned a code number indicating the treatment to be administered to the subject.

7.6. Blind and blind opening

The treatment will be masked, so that neither researcher nor subject itself contains Nyaditum resae® know whether or placebo. Both treatments are physically identical and have the same excipients. The label will also be identical, indicating the source of the study, the numerical identifier of the data subject and related promoter, route of administration and dosage. In an emergency that requires the opening of the blind, the investigator will contact the CRO informed of this and what is the composition of the treatment of the individual. A researcher must justify in writing the reasons that motivate the opening.

8. CHANGING AND MEASURING INSTRUMENTS. DEFINITION AND DESCRIPTION OF THE MEASUREMENT

8.1. Variables studied

8.1.1. Main variables:

• Increase specific Treg cells

• Cell memory one week after their first treatment

• Overall tolerability of treatment Nyaditum resae®.

8.1.2. Secondary variables:

• Local tolerability (gastrointestinal tract).

• tolerability general (vital signs, physical examination, analysis)

• Increase specific Treg cells

• Cell memory to two weeks after their first treatment.

8.2. Procedures for evaluating parameters

8.2.1. Clinical history and physical examination

We collect demographic data of interest to characterize the study population:

- Sex

- Birthdate

- Background and concomitant medication

- BCG Vaccination

- Mantoux or tuberculin skin test (positive if> 5 mm in unvaccinated,> 10 mm in BCG vaccinated)

- Chest X-ray, if the test is positive tuberculin

It collected data baseline physical examination and monitoring:

- Weight (baseline only)

- Height (baseline only)

- Blood Pressure

- Heart rate

- Respiratory

- Temperature axilar

8.2.2. Laboratory analysis

Blood samples obtained at each of the points specified in the schedule of the study (Section 11) after, at least 8 hours of fasting. It quantified the following parameters:

♣ Hematology:
- Hematocrit
- Red blood cells
- Leukocytes
- Hemoglobin
- Platelets

♣ Biochemistry:
- Glucose
- Urea
- Creatinine
- Estimated glomerular filtration rate (calculated by the equation renal diet or modified MDRD) - Ionograma: sodium, potassium
- Total Bilirubin
- Transaminases: SGPT (ALAT), SGOT (ASAT), gamma GT, alkaline phosphatase
- Test urine pregnancy

♣ Microbiology:
- HIV serology

♣ immunogenicity and cytokine profile:
- Presence of Tregs specific memory from PBMCs obtained from whole blood stimulated with PPD and 10 ug / mL for 7 days.
- Cytokine profile.

8.2.3. Daily patient

Data will be collected and tolerability of treatment adherence through a newspaper that fill the patient (see Appendix 7).

We collected the following variables during the four weeks after initiation of treatment:
- Date and time of the seizure treatment
- Faecal stools (number / day)
- Nausea (grade 1, 2 or 3)
- Vomiting (grade 1, 2 or 3)
- Abdominal pain (grade 1, 2 or 3)
- Other adverse effects (grade 1, 2 or 3)

8.3. Chronograma

	**Selection**		**Pre adm.**	**Adm.**	**Follow up**		
Visit	Selection		Basal		S1	S2	S6
Day	-28	-25	0		7	15	42
**Week**	**-4**	**-4**	**0**		**1**	**2**	**6**
Interval (days)	±14		0		±3	±3	±3
Patient Inform consent	X						
Inclusion/exclusion criteria	X		X				
Background	X						
VIH test	X						
Tuberculin test	X[A]	X[B]					
Dx Rx		X[C]					
Urine pregnancy test	X		X				X
Randomization			X				
Tt administration				X			
Physical vital signals	X		X		X	X	X
Haematology& Biochemist	X		X[D]		X	X	X
Immunogenicity			X		X	X	X
Daily Patient				X	X	X	X[E]
Concomitant medication	X		X		X	X	X
Adverse events	X			X	X	X	X

A positive test is accepted in the last 5 years. Accepted test negative in the last 6 months.
B Read the tuberculin test .
C Should be a positive tuberculin skin test .
D do not need to repeat it if the selection has been made in the previous 15 days .
E Collection of daily **patient.**

9. STATISTICAL ANALYSIS

The statistical analysis and data management will be performed by the Foundation Fighting AIDS. The analysis is carried out using SPSS 15.0.

It listed the baseline characteristics of the subjects, separated by groups and tabulates them descriptively. It will be used in testing hypothesis testing because it does not have data on the variation of the intensity of the immune response is expected, which is why this is a pilot study.

The main analysis is based on the description of the variables collected during monitoring, separating groups of treatment. Also describe the temporal profile of the securities in general and separating groups.

The longitudinal study will assess the pattern of evolution of Treg cells and if this is associated with the treatments studied and the different baseline characteristics. To meet this goal will serve mixed models for repeated measures.

The safety profile will be tabulated and comparing the different adverse effects recorded by treatment group. For the description will be used mean (standard deviation), median (interquartile range) or frequencies for comparison between groups will be used t-tests, tests not parametric Xi-square or Fisher's exact test, as appropriate for the distribution of the variable to study.

The analysis will be done in the population under the assumption protocol and intention to treat, which will take the stage Missing = Failure. In the context of this study that individuals have a Missing in Treg is considered that this is the baseline.

It listed all the subjects selected for the study have been excluded from the analysis, descriptive tabular form their baseline characteristics and track information where available. Also describe the reasons for exclusion. If you notice that some baseline clinical or demographic variable influencing determinedly on the assumptions of the study will describe the results and adjusted according to this information. It is considered a significance level of 5% and, if necessary, make changes to the data. The missing data is not specifically describe the evidence that might suggest a loss is not random (ie, the lost value rise was conditioned by this parameter take value); Loss for the other cases at random "or" completely random "- do not take any action, because the lost data have the same distribution that the observed data or the data observed conditional value the other covariates registered.

9.1. Description of the sample size

The show is based on the calculation of the power to see the factors associated with response in a regression model . To assess whether there is a relationship between the production of cell -specific Treg memory cells and the intervention group , taking into account latency TBC , using a regression model and assuming that the initial explanatory variables explain 20% the variability of response and the final multivariate model could explain up to 40% of the response , you need to include a minimum of 41 individuals in the study had a 80% power to detect differences studied with a level of significance of 5%. This sample size is smaller than the finally included in the study, allow this margin to cope with slightly different results of which have been accepted as plausible .

10. ETHICAL ASPECTS

This study will be conducted following the ethical principles contained in the Declaration of Helsinki, in October 2008. The protocol will be assessed by the Clinical Research Ethics Committee of the Hospital Germans Trias i Pujol. The participation of researchers in this study is free, voluntary and independent.

10.1. Risk-benefit assessment for research subjects

It is expected that the subject may have no benefit for individual participation in this clinical trial. The overall objective of the study is the effect of Nyaditum resae® on immunity, which could favor the development of protection against tuberculosis. Anyway, participation in this trial can be of great use to a better understanding of how this probiotic. Potential adverse effects of Nyaditum resae® are slight alterations of intestinal transit. The blood can cause local discomfort or bruising in the area as a result of the puncture. The tuberculin skin test may cause local transient discomfort.

10.2. Information sheet and consent form

The investigator must inform the subjects of the nature, duration and purpose of this study, and also for all the inconveniences and obstacles, within what is reasonable to expect. In addition it will provide written information on the subject (see Appendix 5). Volunteers participants must have legal capacity to give consent and exercise their freedom of choice. Written consent be obtained before including the subject in the study (Appendix 6).

10.3 . Data confidentiality

The sponsor of the study and researchers ensure the confidentiality of data subjects and shall ensure that at all times fulfilled the provisions of Law 15/1999 on Protection of Personal Data . The processing of the data that the developer of the study to collect during the study will be subject to the law as to data protection. The subjects identified in records only the code number . The subject is no guarantee anonymity and they should be informed that all communication will take place between him and the investigator , and the sponsor of the study. If the person decides to withdraw consent to participate in this study, no new data will be added to the database and may require the destruction of all previously retained identifiable samples to avoid conducting new an

11. PRACTICAL CONSIDERATIONS

11.1. Work Plan

The subjects are recruited through an informative poster (Annex IX) which will hang on the Teaching Unit and the Germans Trias i Pujol Hospital Germans Trias i Pujol, as well as volunteers registered mail sent to the Base Data Sans Volunteer Service Clinical Pharmacology (Annex X). There will also be a briefing at the hospital. Those interested to attend Multipurpose Clinical Research Unit. There, you will be informed of the nature and duration of the study. After accepting their participation (informed consent), an identifier assigned to the subject. In order to identify other individuals who participate in clinical trials phase, participants will be on the Register of Clinical Trials Voluntary Health Department, according to the usual procedure. There will be the visit of selection, which consists of a clinical evaluation and a blood sample to check the criteria. If necessary (see diagram Annex VIII) will be held tuberculin skin test and must mention the subject in three days to read. If the tuberculin test is negative and meet the other criteria, it will mention the subject for inclusion in the study (baseline). If the skin test is positive, a chest X-ray will be done. If you rule out tuberculosis and comply with other criteria, it will mention the subject for inclusion in the study (baseline). If the chest radiograph awake active tuberculosis, the subject will be discarded by the test and derive emergencies. If you visit during the selection occurs any clinically relevant finding, researchers will examine the appropriateness of the subject included in the study, but in any case, be derived from the GP.

At baseline , there will be detailed procedures on schedule , including a physical examination and a blood sample for immunogenicity , among others. By randomization assigned a number to the subject kit containing masked treatment . Directions will continue and will mention the subject to the following visits , which will be at weeks 1, 2 and 6. In these visits will be detailed procedures on schedule . Subjects will fill in a diary for 4 weeks after starting treatment

11.2. Monitoring reports and final

It is planned to make a single final report of the study can be seen as publications arising from the final report of the study results . These documents will be forwarded to the Ethics Committee , as well as any issues that may arise.

11.3. Dissemination of results

It is planned to disseminate the results of this study health interest in national and international conferences and publications in one or several English speaking (" peer reviewed ") .

12. What is a probiotic? Regulatory issues

Probiotics are defined as nutritional supplements containing viable pathogenic microorganisms that confer a health benefit on the host. Its use has been widely promoted, suggesting that standards play an important role in the immune, digestive and respiratory (1). Recently it has been shown that probiotics do not need to maintain their viability to induce their beneficial effects. This aspect is very interesting because it facilitates standardization of such products, increases stability and also can increase the range of organisms that can be considered probiotics. Also, since there is always the risk that the live probiotics can generate some kind of pathology, making it viable probiotics are much safer (2).

The bacilli of Mycobacterium fortuitum complex are usually dust or water (3-6). Specifically in the tap water can be found in concentrations between 10^2-10^3 and up to 105 colony forming units per liter (UFCs / L) (7); UFCs and between 20-2500 / L water pool (8). This means that Mycobacterium fortuitum is so common in our diet and therefore is in contact with our intestinal mucosa, although no one knows how often and what effect causing population level.

The European Food Safety Authority (EFSA) states that can be "health claims" relating to the consumption of food or any of its components such as vitamins and minerals, fiber and probiotic bacteria. Specifically, Article 14 of Regulation (EC) No. 1924/2006 authorized health claims, claiming, suggesting or giving to understand that the consumption of a food category, a food or one of its constituents reduce a significant risk factor for onset of human disease.

Thus probiotics, considering themselves part of a food, there is no legislation in the European Union to regulate its use in human trials conducted, except that it must comply with the ethical requirements internationally accepted as is reviewed by the Ethics Committee concerned. Keep in mind that, being a food can never be referred to the treatment, prevention or treatment of a human disease, as established in Royal Decree 1334/1999. If the product appears with properties for treating or preventing disease in human beings, would be under the definition of a drug under section 2.1. Real Decreto 1345/2007, which regulates the procedure for the authorization, registration and conditions of dispensation of medicines for human use manufactured industrially, and should be subject to specific legislation that regulates them, including Royal Decree 223 / 2004. EC Regulation 1924/2006 applies to all nutrition and health claims made in commercial communications, including advertising campaigns including cabbage • teaching and promotional campaigns, such as those sponsored fully or partially by the authorities Public. However, you need not apply to statements made in non-commercial communications, such as dietary advice or guidance provided by authorities or public health agencies or non-commercial communications and information in the press and scientific publications . Then attach the responses made by the Spanish Food Safety Agency concerning the execution of clinical trials with food.

Referències

1. Pineiro M, Stanton C. Probiotic bacteria: legislative framework-- requirements to evidence basis. The Journal of nutrition. 2007;137(3 Suppl 2):850S-3S.
2. Adams CA. The probiotic paradox: live and dead cells are biological response modifiers. Nutrition research reviews. 2010;23(1):37-46.
3. Martin Casabona N, Rossello Urgell J. [Environmental mycobacteria in Spain: isolations in the 1976-1996 period]. Medicina clinica. 2000;115(17):663-70.
4. Brown TH. The rapidly growing mycobacteria--Mycobacterium fortuitum and Mycobacterium chelonei. Infection control : IC. 1985;6(7):283-8.
5. Jin BW, Saito H, Yoshii Z. Environmental mycobacteria in Korea. I. Distribution of the organisms. Microbiology and immunology. 1984;28(6):667-77.

6. Imwidthaya P, Suthiravitayavaniz K, Phongpanich S. Mycobacterium other than tubercle bacilli in various environments in Bangkok. Journal of the Medical Association of Thailand = Chotmaihet thangphaet. 1989;72(6):317-20.
7. Fischeder R, Schulze-Robbecke R, Weber A. Occurrence of mycobacteria in drinking water samples. Zentralblatt fur Hygiene und Umweltmedizin = International journal of hygiene and environmental medicine. 1991;192(2):154-8.
8. Leoni E, Legnani P, Mucci MT, Pirani R. Prevalence of mycobacteria in a swimming pool environment. Journal of applied microbiology. 1999;87(5):683-8.

GOBIERNO DE ESPAÑA | MINISTERIO DE SANIDAD, SERVICIOS SOCIALES E IGUALDAD | agencia española de seguridad alimentaria y nutrición

Agencia Española de Seguridad Alimentaria y Nutrición

Mapa del Sitio
Contacto
Imprimir

Buscar

Contacto con la Agencia

Pregunta

Ensayos Clínicos con alimentos.

Respuesta

Actualmente no existe legislación en España ni en Europa que regule esta materia.

Únicamente están regulados los ensayos clínicos con medicamentos mediante el RD 223/2004, de 6 de febrero. No obstante, los ensayos realizados con humanos deben cumplir requisitos éticos aceptados internacionalmente, y por lo tanto deberán ponerse en contacto con las autoridades competentes hospitalarias de la Comunidad Autónoma donde se realicen los ensayos, para que les indiquen los trámites a seguir en la aprobación de los requisitos éticos de los estudios.

Finalmente, le informo de que si el objetivo final de la realización de estos estudios, es la presentación de una solicitud de declaraciones saludables en los alimentos, la Agencia Europea de Seguridad Alimentaria (EFSA), publicó la guía "Scientific and technical guidance for the preparation and presentation of the application for authorisation of a health claim", que recoge orientaciones sobre los aspectos metodológicos y éticos que deben cumplir los ensayos de intervención en humanos con alimentos, para poder ser considerados válidos a la hora de sustentar las solicitudes de declaraciones saludables.

Para mayor aclaración sobre el trámite a seguir para la presentación de solicitudes de declaraciones saludables de los alimentos, puede consultar la información disponible sobre este tema que aparece en la página web: http://www.aesan.mspsi.es/AESAN/web/cadena_alimentaria/detalle/normativa_aplicacion.shtml
17.03.2011

Agencia Española de Seguridad Alimentaria y Nutrición

Mapa del Sitio
Contacto
Imprimir

Buscar

Contacto con la Agencia

Pregunta

¿Están regulados los ensayos clínicos con alimentos?

Respuesta

Únicamente están regulados los ensayos clínicos con medicamentos mediante el RD 223/2004, de 6 de febrero, sin que exista normativa que regule los ensayos de intervención en humanos con alimentos.
Por lo tanto no hay establecidos trámites administrativos por parte de la AESAN previos a la realización de un ensayo de intervención en humanos con alimentos, y que no recaiga en el ámbito de los medicamentos.
No obstante, se debe tener en cuenta que cualquier ensayo realizado con humanos, bien sea con medicamentos o con alimentos, debe cumplir requisitos éticos aceptados internacionalmente, como es la revisión por el comité de ética correspondiente.
Por otro lado, el uso de un producto alimenticio nunca puede referirse a la curación, prevención o tratamiento de enfermedades humanas, tal y como se establece en el art. 4.1.d) del Real Decreto 1334/1999, por el que se aprueba la Norma general de etiquetado, presentación y publicidad de los productos alimenticios.
En este sentido, si un producto se presenta con propiedades para el tratamiento o prevención de enfermedades en seres humanos, estaría bajo la definición de medicamento, según el art. 2.1 del Real Decreto 1345/2007, por el que se regula el procedimiento de autorización, registro y condiciones de dispensación de los medicamentos de uso humano fabricados industrialmente, y tendría que someterse a la legislación específica que los regula, incluyendo el citado Real Decreto 223/2004.
No obstante, los productos alimenticios podrán realizar cualquier declaración nutricional o de propiedades saludables, que cumpla con los criterios establecidos en el Reglamento 1924/2006, de 20 de diciembre relativo a las declaraciones nutricionales y de propiedades saludables en los alimentos.
Por lo tanto, teniendo en cuenta todo lo anterior, la población diana a la que pueden ir dirigidas las citadas declaraciones nutricionales o de propiedades saludables, debe ser la población general sana, y no los pacientes de alguna enfermedad humana.
Para obtener más información sobre las declaraciones nutricionales y de propiedades saludables en los alimentos, se puede consultar el siguiente link de nuestra página web:
http://www.aesan.mspsi.es/AESAN/web/cadena_alimentaria/detalle/tipos_declaraciones.shtml

13. Adverse events

13.1. Definition Adverse event (AE):

Any adverse medical event that may present a clinical research subject, whom he has administered treatment and should not necessarily have a causal relationship with this treatment. Serious adverse events (SAE):

♣ Produce death

♣ Endangers life

♣ Requires or prolongs hospitalization

♣ Produce a disability / persistent or significant disability and an abnormality or birth defect.

♣ It is an event not included in the preceding paragraphs, but in the opinion of the investigator, may jeopardize the subject or may require intervention to prevent any of the above mentioned results.

Unexpected adverse events:

AE that is related to study treatment, the nature or intensity that does not match the information available product (Technical Manual for marketed products or products not traded by the investigator). Unexpected serious adverse reaction (Mane, Susara) is that SAE related to study treatment, the nature and intensity of which do not match the available information of the product (or products marketed by Technical Manual investigator for products not marketed).

13.2. Description of accountability criteria

The causal relationship established by the algorithm Spanish Pharmacovigilance System, which defines the following categories:

Short:
♣ There is a plausible sequence in connection with the administration or treatment with tissue or plasma levels of it.
♣ The demonstration coincides with the observed pattern of adverse events known treatment involved.
♣ It can not be explained by concurrent disease or other treatments or chemicals.
♣ The response to the withdrawal should be clinically plausible. So, better to discontinue the administration of treatment.
♣ The process of recapitulation must be positive.

Probable:
♣ There is a sequence reasonable exposure treatment.
♣ The demonstration coincides with the observed pattern of adverse events known treatment involved.
♣ It is unlikely that it can be attributed to intercurrent disease or other treatments or chemicals.
♣ After being retired clinical treatment follows a sequence reasonable.
♣ Improved administration to discontinue treatment.
♣ reprise not required to complete this definition.

Possible :

♣ There is a sequence reasonable exposure treatment.

♣ coincides with the known pattern of adverse events .
♣ It may be due to the clinical condition of the subject or chemicals or other treatments administered concomitantly .
♣ information on the withdrawal may not exist or be confused .

unlikely :

♣ A clinical event , including laboratory test abnormalities , compared with a temporal relationship to the administration of treatment that makes unlikely causality and the other treatments , chemicals, or diseases undercurrent provide plausible explanations .

Not related :

Does not meet any of the criteria mentioned ..

13.3. Procedure Notification of adverse events

Researcher

The investigator immediately notify the promoter serious adverse events (EAG) (see section 13.1).

The notification will be done in the first 24 hours of their knowledge by completing the form of notification of serious adverse events (Annex II of this protocol) or adverse events (Annex I, within the QRD), which sent by fax (93 465 76 02) or by email to monitor the study.

We recorded all adverse events regardless of causality attributed in the form of description of adverse events. This form is in the QRD each subject in the study (Appendix I).

The collection from EA will be conducted by the team of researchers of the test, indicating the time of occurrence specify expressed in the minimum possible time unit if it is serious or not serious in the case of be related, if you expected or unexpected, its intensity (grade 1, 2, 3), the measures taken (none, symptomatic treatment, temporary or permanent discontinuation of study treatment), evolution (solved or not) and accountability (causal relationship) to be used on the criteria defined in section 13.2.

The intensity will be determined by the investigator using the following approach :

♣ 1 = Mild . Event causing interference • zero or minimal functional activities .
♣ 2 = Moderate . Event causing more than minimal interference in functional activities .
♣ 3 = intense . Event causing inability to perform functional activities .

the developer

The sponsor shall notify the Ethics Committee any important information security products research and Mane associated with product research.

From the moment that the developer was aware of suspected serious adverse events , the period of notice shall be:
♣ 15 days
♣ 7 days if adverse reactions have caused death or seriously endangered the life of the subject. Over eight days to complete all the information.

The developer will maintain detailed records of all adverse events notified by the researchers . All adverse events reported are tabulated for the final report of the study.

APPENDIX I: DATA COLLECTION NOTEBOOK

ANNEX II: SAE FORM

ANNEX III: Ethical committee agreement

ANNEX IV: Technical file of the product

ANNEX V: INFORMATION FILE FOR THE PARTICIPANTS

ANNEX VI: INFORMED CONSENT

ANNEX VII: PATIENTS DIARY

ANNEX VIII: INCLUSION DIAGRAM

ANNEX IX: INFORMATIVE ADVERTISEMENT

Wanted seniors to participate in a clinical trial paid a probiotic oral administration . Interested / please contact:

- Dr. Pere Joan Cardona, T. 60 629 61 99 mail pjcardona@manremyc.cat

- Clinical Pharmacology Service , T. 93 497 88 65 - Multipurpose Clinical Research Unit , T. 93 497 84 88

May not participate Pregnant or lactating women or people with immunosuppressive therapy , immunodeficiency , tuberculosis or HIV positive. Neither intolerant people can participate mannitol or sucrose .

ANNEX X : MAIL INFORMATION TO VOLUNTEERS

Dear volunteer, We will contact you to see if you would be interested in participating in a clinical trial paid a probiotic oral administration.

May not participate Pregnant or lactating women or individuals with immunosuppressive therapy , immunodeficiency , tuberculosis or HIV positive.

Neither intolerant people can participate mannitol or sucrose .

If you have any questions or are interested in participating , please contact us.

Sincerely,

UPIC
