# Supplementary material for: Pilot, double-blind, randomized, placebo-controlled clinical trial of the supplement food Nyaditum resae® in adults with or without latent TB infection: Safety and immunogenicity
Source: PLoS One. 2017 Feb 9;12(2):e0171294. doi: 10.1371/journal.pone.0171294 (PMC5300153; doi:10.1371/journal.pone.0171294)
Supplement: S3 File — (PDF) [file pone.0171294.s003.pdf]

ID PACIENTE |__|__|__|__|__|__|

NYADATREG

ENSAYO CLÍNICO PILOTO, DOBLE-CIEGO, ALEATORIZADO, CONTROLADO CON PLACEBO PARA EVALUAR LA TOLERABILIDAD Y INMUNOGENICIDAD DEL PROBIÓTICO NYADITUM RESAE® ADMINISTRADO EN ADULTOS CON O SIN INFERCCIÓN TUBERCULOSA LATENTE.

Versión 1, 22 de Octubre de 2013

Cuaderno Recogida Datos

Promotor:
Manresana de Micobacteriologia, SL.
C/ Urgell nº 26,1
08240-Manresa

ID PACIENTE |__|__|__|__|__|__|

Datos demográficos

Fecha de nacimiento: ___ /___ /______

Fecha de obtención del consentimiento: ___ /___ /______

Sexo: ☐ Hombre ☐ Mujer

Test VIH

Fecha de extracción: ___ /___ /______ Resultado: ☐ Positivo ☐ Negativo

Test embarazo en orina

Fecha de extracción: ___ /___ /______, Resultado: ☐ Positivo ☐ Negativo

Antecedentes

¿Tiene el paciente alguna enfermedad actual o previa relevante? ☐ Sí ☐ No

Procesos del último año ***(Si la respuesta es Si, anótelas debajo)***

Enfermedades relevantes	Conocido desde	¿Continua presente? SÍ NO
		☐ ☐
		☐ ☐
		☐ ☐
		☐ ☐
		☐ ☐

ID PACIENTE |__|__|__|__|__|__|

Tuberculosis

Vacunación BCG ☐ Si → ¿Tiene cicatriz? ☐ Si ☐ No
☐ No

Test Tuberculina ☐ No realizado → (1)

☐ Realizado → (2)

(1)

Fecha realización: ___ /___ /______ Fecha lectura: ___ /___ /______

Resultado (mm) ________

Se considera: ☐ Positivo → (3)

☐ Negativo →"Se puede incluir como ITBL negativo"

(2)

¿El resultado fue? ☐ Negativo → ¿Hace menos de 6 meses?

☐ Si → "Se puede incluir como ITBL negativo"

☐ No → "Se debe volver a realizar el test" (1)

☐ Positivo → ¿Hace más de 5 años?

☐ Si → "Se debe volver a realizar el test" (1)

☐ No → (3)

(3)

Radiografía de tórax: Fecha realización: ___ /___ /______

¿Presenta tuberculosis? ☐ Si →"No puede participar en el estudio. Según el protocolo, el candidato debe derivarse a Urgencias"

¿Presenta tuberculosis? ☐ No →"Se puede incluir como ITBL positivo"

ID PACIENTE |__|__|__|__|__|__|

Criterios de inclusión / exclusión

Criterios de inclusión	SI	NO
1. Firma del consentimiento informado.	☐	☐
2. Mujer u hombre con edad igual o superior a 18 años.	☐	☐
3. Disponibilidad para cumplir los requerimientos del protocolo.	☐	☐

Criterios de exclusión	SI	NO
1. Pacientes infectados por el VIH.	☐	☐
2. Inmunodeficiencias conocidas.	☐	☐
3. Mujeres embarazadas o en período de lactancia materna.	☐	☐
4. Tuberculosis activa.	☐	☐
5. Participación en otro ensayo clínico.	☐	☐
6. Intolerancia a manitol o sacarosa.	☐	☐
7. Administración crónica de: metotrexato, azatioprina, ciclofosfamida, corticosteroides orales y otras terapias inmunosupresoras o inmunomoduladores.	☐	☐
8. Administración de productos sanguíneos o derivados de la sangre durante los 6 meses previos a la aleatorización.	☐	☐
9. Detección por parte del investigador de falta de conocimiento o voluntad para participar y cumplir con todos los requerimientos.	☐	☐
10. Cualquier otro criterio que el investigador piense que pueda comprometer el cumplimiento del protocolo o influenciar significativamente en la interpretación o en los resultados de los efectos del probiótico.	☐	☐

Aleatorización

Fecha de aleatorización: ___ /___ /______

Número de kit asignado al sujeto: __________ (según tabla de aleatorización para ITBL + o -)

¿Se requiere sustituir el kit? ☐ No

☐ Si → "Llame a la monitora, T. 93 497 84 14"

Motivo de sustitución: (texto largo)

Fecha de sustitución: ___ /___ /______

Nuevo número de kit asignado al sujeto: __________

ENVIAR CONFIRMACIÓN DE ALEATORIZACIÓN POR MAIL A MONITORA (rescrig@flsida.org) Y A LAS INVESTIGADORAS.

ID PACIENTE |__|__|__|__|__|__|

Visita médica: signos vitales y exploración física

	Basal (día 0)		Semana 1		Semana 2		Semana 6	
Fecha exploración	___ /___ /______		___ /___ /______		___ /___ /______		___ /___ /_____	
Talla (m)								
Peso (Kg)								
TAS (mmHg)								
TAD (mmHg)								
Frec. Cardiaca (puls/min)								
Frec. Respiratoria (respiraciones/min)								
Exploración física	☐	Normal	☐	Normal	☐	Normal	☐	Normal
	☐	Anormal	☐	Anormal	☐	Anormal	☐	Anormal
Si la exploración física es anormal, especificar								
¿Existe algún hallazgo relevante?	☐ No<br>☐ Si → "Inclúyalo como condición basal en "antecedentes""		☐ No<br>☐ Si → "Inclúyalo como acontecimiento adverso""		☐ No<br>☐ Si → "Inclúyalo como acontecimiento adverso""		☐ No<br>☐ Si → "Inclúyalo como acontecimiento adverso""	

Analítica

Hematología

	Basal (día 0)	Semana 1	Semana 2	Semana 6
Fecha de extracción:	___ /___ /______	___ /___ /______	___ /___ /______	___ /___ /______
Leucocitos (x10^9/L)				
Hematíes (x10^{12}/L)				
Hemoglobina (g/dL)				
Hematocrito (%)				
Plaquetas (x10^9/L)				
¿Existe algún hallazgo clínicamente relevante?	☐ No<br>☐ Si → "Inclúyalo como condición basal en "antecedentes""	☐ No<br>☐ Si → "Inclúyalo como acontecimiento adverso""	☐ No<br>☐ Si → "Inclúyalo como acontecimiento adverso""	☐ No<br>☐ Si → "Inclúyalo como acontecimiento adverso""

ID PACIENTE | 3| 0|0 |__|__|__|__|__|__|

Bioquímica sérica

	Basal (día 0)	Semana 1	Semana 2	Semana 6
Fecha de extracción	___ /___ /______	___ /___ /______	___ /___ /______	___ /___ /______
Glucosa (mg/dL)				
Urea (mg/dL)				
Creatinina (mg/dL)				
MDRD				
Sodio (mmol/L)				
Potasio (mmol/L)				
Bilirrubina total (mg/dL)				
Fosfatasa alcalina (U/L)				
GOT / AST (U/L)				
GPT / ALT (U/L)				
GGT (U/L)				
¿Existe algún hallazgo clínicamente relevante?	☐ No<br>☐ Si → "Inclúyalo como condición basal en "antecedentes""	☐ No<br>☐ Si → "Inclúyalo como acontecimiento adverso""	☐ No<br>☐ Si → "Inclúyalo como acontecimiento adverso""	☐ No<br>☐ Si → "Inclúyalo como acontecimiento adverso""

ID PACIENTE | 3| 0|0 |__|__|__|__|__|__|

FINALIZACIÓN DEL ESTUDIO

Fecha de finalización: ___ /___ /______

¿Se trata de una finalización prematura? ☐ Sí ☐ No

En caso afirmativo, por favor, indique el motivo:

☐ Pérdida de seguimiento

☐ Muerte → Fecha de la muerte: ___ / ___ / ______

☐ Proceso o enfermedad intercurrente que en opinión del investigador requiere la retirada.

☐ Desviación o incumplimiento del protocolo

☐ Abandono voluntario (retirada de consentimiento)

☐ Acontecimiento adverso

¿El sujeto ha tomado menos de 5 dosis de tratamiento? ☐ Sí ☐ No

ENVIAR CONFIRMACIÓN DE FINALIZACIÓN PREMATURA POR MAIL A MONITORA (rescrig@flsida.org) Y A LAS INVESTIGADORAS.

ID PACIENTE | 3| 0|0 |__|__|__|__|__|__|

Tratamiento concomitante

Nombre genérico de la medicación	Dosis diaria	Vía (*)	Fecha de inicio	Fecha de fin	Indicación

(*) Vía de administración:

- PO = vía oral
- IH = inhalación
- VR = vía rectal

TO = tópica
SC = subcutánea

IM = intramuscular
IV = intravenosa

ID PACIENTE | 3| 0|0 |__|__|__|__|__|__|

Acontecimientos adversos INCLUIR DEFINICIONES EN EL CRD ELECTRÓNIC (SIGUIENTE PÁGINA)

Descripción del AA	Fecha del AA		Intensidad del AA	Terapia para el AA	Actitud tomada con el tratamiento en estudio	¿Fue grave?	Relación causal con la medicación en estudio y esperabilidad	
	Inicio	Resolución	1: Leve<br>2: Moderado<br>3: Intenso	0: No<br>1: Si<br>(anotar en tratamiento concomitante)	1: Ninguna<br>2: Interrupción temporal<br>3: Interrupción definitiva	0:No<br>1: Si<br>Si la respuesta es sí, rellenar el formulario de SAE	1: Definitiva<br>2: Probable<br>3: Posible<br>4: Improbable<br>5: No relacionado	0: Esperado<br>1: Inesperado
1.								
2.								
3.								
4.								
5.								
6.								

Descripción del AA (si es necesario), utilizando el número de línea de arriba.

EN CASO DE SAE, ENVIAR NOTIFICACIÓN POR MAIL A LA MONITORA (rescrig@flsida.org) Y A LAS INVESTIGADORAS.

ID PACIENTE | 3| 0|0 |__|__|__|__|__|__|

DEFINICIONES

Intensidad del AA

Leve: Acontecimiento que causa una interferencia nula o mínima en las actividades funcionales.
Moderado: Acontecimiento que causa más que una interferencia mínima en las actividades funcionales.
Intenso: Acontecimiento que causa incapacidad para realizar actividades funcionales.

Relación causal con la medicación en estudio y esperabilidad

Definitiva

- Existe una secuencia temporal plausible en relación con la administración del tratamiento o con los niveles plasmáticos o tisulares del mismo.
- La manifestación observada coincide con el esquema de acontecimientos adversos conocidos del tratamiento implicado.
- No se puede explicar por enfermedad concurrente o por otros tratamientos o sustancias químicas.
- La respuesta a la retirada tiene que ser clínicamente plausible. O sea mejora al interrumpir la administración del tratamiento.
- El proceso de reexposición tiene que ser positivo.

Posible

- Existe una secuencia temporal razonable a la exposición del tratamiento.
- Coincide con el esquema de reacciones adversas conocidas.
- Puede ser debida a la situación clínica del sujeto o a otros tratamientos o sustancias químicas administradas concomitantemente.
- La información sobre la retirada puede no existir o ser confusa.

Improbable:

- Un acontecimiento clínico, incluidas anomalías en las pruebas de laboratorio, con una relación temporal respecto a la administración del tratamiento que hace improbable la relación de causalidad, y en el que otros tratamientos, sustancias químicas o enfermedad intercurrente proporcionan explicaciones plausibles.

No relacionado

No cumple ninguno de los criterios mencionados.

Acontecimiento Adverso Grave

1: Produce la muerte.
2: Pone en peligro la vida.
3: Precisa de ingreso hospitalario o lo prolonga.
4: Produce una discapacidad / incapacidad persistente o importante, así como una anomalía o defecto congénito.
5: Es un acontecimiento no incluido en los anteriores puntos, pero que, a criterio del investigador, puede poner en peligro al sujeto o puede requerir intervención para prevenir cualquiera de los anteriores resultados mencionados.
