# Supplementary material for: Pilot, double-blind, randomized, placebo-controlled clinical trial of the supplement food Nyaditum resae® in adults with or without latent TB infection: Safety and immunogenicity
Source: PLoS One. 2017 Feb 9;12(2):e0171294. doi: 10.1371/journal.pone.0171294 (PMC5300153; doi:10.1371/journal.pone.0171294)
Supplement: S4 File — (PDF) [file pone.0171294.s004.pdf]

MANREMYC

Manresana de Micobacteriologia sl.

NYADITUM RESAE ® (NR)

PRODUCT DOSSIER

INDEX

1. BACKGROUND & STATE OF ART..2

1.1. Introduction:..2

1.2. The inflammatory component of TB and the origin of Nyaditum resae® 3

1.3. Nyaditum resae® 4

1.4. References 4

2. BRIEF SUMMARY OF THE NONCLINICAL & CLINICAL DATA……………………...…………………….6

3. PRE-CLINICAL EXPERIMENTS CONDUCTED WITH *NYADITUM RESAE*®: PROTOCOL SUMMARY & RESULTS...9

3.1. EXPERIMENT #1 (nyada #18)………………………………………. ………………………….11

3.1. EXPERIMENT #2 (nyada #16-prophylaxis)………………………………………………..…...13

3.1. EXPERIMENT #3 (nyada #16-therapeutic)……………………………………………..…...15

3.1. EXPERIMENT #4 (nyada #19)…………………………………………………………………...17

3.1. EXPERIMENT #5 (nyada #21)………………………………………….………………….19

3.1. EXPERIMENT #6 (nyada #23)………………………………………….……….……….21

3.1. EXPERIMENT #7 (nyada #25)………………………………………….………………….....23

ANNEX 1:PROTOCOL OF THE *Nyaditum resae*® CT, NYADATREG. SYNOPSIS…….........................25

1. BACKGROUND & STATE OF ART

1.1. Introduction:

The high incidence of tuberculosis (TB) is still a striking problem with important health and finantial consequences. One and a half million people die every year worldwide, there are 10 million cases of disease and 100 million of new cases of *M.tuberculosis* infection. Moreover, the prevalence of Multi-Resistant to Drugs (MDR) TB is of 700.000 patients, even if this figure grows every year with 100.000 people [1].

It is estimated that one third of the humankind suffers from Latent Tuberculosis Infection (LTBI). Only a 5% of these people develop the active disease within the first 10 years after being infected, the remaining 95% being latently infected (figures 1 & 2) if not diagnosed by specific tests [2].

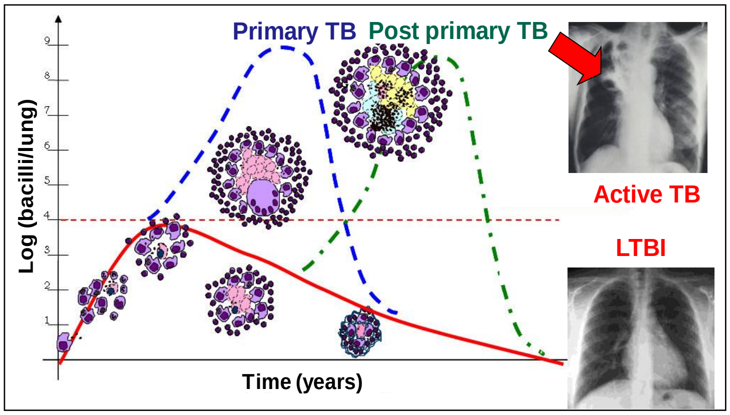

Figure 1. Evolution of TB infection in humans. Once the immunological specific response is triggered, the infection can evolve towards active disease (primary TB) or towards LTBI, which can become a post-primary TB with a probability which decreases with time.

Preventing TB is very difficult. Its infection is due to *Mycobacterium tuberculosis*, a microorganism which is transmitted through the air. There is no well-identified risk to become infected, and no prophylactic vaccine exists able to avoid infection. Since 15 years ago, lots of efforts devoted to substitute the only existing vaccine (Bacil de Calmette Guerin, BCG) have been done, and there are several candidates [3,4]. All these vaccine candidates are seeking an important antigen, able to trigger a secreting IFN-γ Th1 response to activate the infected macrophages and to destroy the bacilli inside them [3]. Even if a decrease in the bacillary load is achieved in the experimental models, this has not been correlated with protection against infection and disease [5]. In fact, the BCG itself, administered from 1927 to newborn babies,

confers protection to acquire mortal forms of TB (meningeal and miliar), but has not the ability to avoid infection [6].

This is because even if having the best immune response, once the bacilli have entered the pulmonary alveoli, has been phagocyted by an alveolar macrophage and has began to grow, the infection is already set, and thus the reinfection is possible, as described by the Dynamic Hypothesis [7].

However, the most crucial issue in TB is the induction of active disease in LTBI patients (figure 2), and yet no theory can convincingly explain this phenomenon.

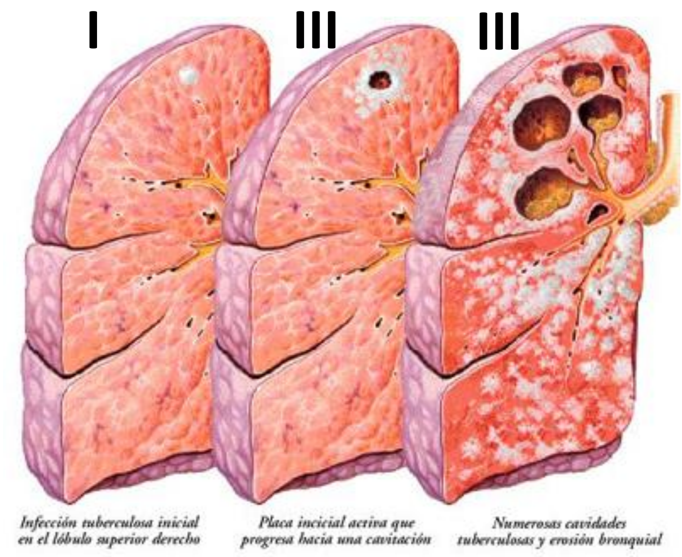

Figure 2:

The development of active TB in humans is pictured. In a 10% of the patients, a small lesion (I), will grow and even generate a cavity, developing disease (II i III). (From Netter Images.com)

1.2. The inflammatory component of TB and the origin of Nyaditum resae®

The liquefaction (the necrotic tissue becoming liquid) is considered a crucial point in the disease development, and the Experimental Tuberculosis Unit (UTE) has been lately focused on developing and characterizing different experimental animal models able to mimic it [8]. As a result of their studies on minipigs [9] and mice (using the Kramnik mouse strain [10]), they described the liquefaction being possible because of the confluence of several lesions with a high proportion of neutrophiles (i.e. a high component of inflammation) [10]. These results were further confirmed by demonstrating the administration of low-dose aspirin or ibuprofen increase the animals' survival in a statistically significant way [11]. Consequently, the UTE started to work

on generating *M.tuberculosis*-specific oral tolerance and developed the product NR. Tolerance is described as “any mechanisms by which the prevention, supression, or reversion of a potentially detrimental immune response is done” [12]. Oral tolerance is a reknown mechanism to induce tolerance to prevent hipersusceptibility against food proteins or bacterial antigens from the intestinal flora [12-14].

1.3. Nyaditum resae®

Nyaditum resae® is based on inactivated *M.fortuitum* strain *Manresa*, a mycobacteria isolated from nature and non-pathogenic. The administration of Nyaditum resae® achieves a maintained anti-inflammatory response able to let the lesions to encapsulate, avoiding their particular growth and further coalescence (thus the liquefaction). Its mechanism of action is the generation of Regulatory T cells (Treg), which action is to modulate the immune response when the Th17 response is exagerated [15-18].

1.4. References

1. Eurosurveillance editorial t. ECDC and WHO/Europe joint report on tuberculosis surveillance and monitoring in Europe. Euro surveillance : bulletin Europeen sur les maladies transmissibles = European communicable disease bulletin. 2013;18(12).
2. Cardona P, Ruiz-Manzano J. On the nature of Mycobacterium tuberculosis-latent bacilli. The European respiratory journal. 2004;24(6):1044-51.
3. Brennan MJ, Thole J. Tuberculosis vaccines: a strategic blueprint for the next decade. Tuberculosis. 2012;92 Suppl 1:S6-13.
4. Ottenhoff TH, Kaufmann SH. Vaccines against tuberculosis: where are we and where do we need to go? PLoS pathogens. 2012;8(5):e1002607.
5. Ottenhoff TH, Ellner JJ, Kaufmann SH. Ten challenges for TB biomarkers. Tuberculosis. 2012;92 Suppl 1:S17-20.
6. Andersen P, Doherty T. The success and failure of BCG - implications for a novel tuberculosis vaccine. Nat Rev Microbiol. 2005;3(8):656-62.
7. Cardona PJ. A dynamic reinfection hypothesis of latent tuberculosis infection. Infection. 2009;37(2):80-6.
8. Guirado E, Amat I, Gil O, Díaz J, Arcos V, Caceres N, et al. Passive serum therapy with polyclonal antibodies against Mycobacterium tuberculosis protects against post-chemotherapy relapse of tuberculosis infection in SCID mice. Microbes and infection / Institut Pasteur. 2006;8(5):1252-9.
9. Gil O, Díaz I, Vilaplana C, Tapia G, Díaz J, Fort M, et al. Granuloma encapsulation is a key factor for containing tuberculosis infection in minipigs. PloS one. 2010;5(4):e10030.
10. Yan BS, Kirby A, Shebzukhov YV, Daly MJ, Kramnik I. Genetic architecture of tuberculosis resistance in a mouse model of infection. Genes and immunity. 2006;7(3):201-10.

10. Marzo E, Vilaplana C, Tapia G, Diaz J, Garcia V, Cardona PJ. Tuberculosis. Damaging role of neutrophilic infiltration in a mouse model of progressive tuberculosis. Available online 19 September 2013, http://dx.doi.org/10.1016/j.tube.2013.09.004
11. Vilaplana C, Marzo E, Tapia G, Diaz J, Garcia V, Cardona PJ. Ibuprofen therapy resulted in significantly decreased tissue bacillary loads and increased survival in a new murine experimental model of active tuberculosis. The Journal of infectious diseases. 2013;208(2):199-202.
12. Weiner HL. Induction and mechanism of action of transforming growth factor-beta-secreting Th3 regulatory cells. Immunological reviews. 2001;182:207-14.
13. Yoshida T, Hachimura S, Kaminogawa S. The oral administration of low-dose antigen induces activation followed by tolerization, while high-dose antigen induces tolerance without activation. Clinical immunology and immunopathology. 1997;82(3):207-15.
14. Zhang X, Izikson L, Liu L, Weiner HL. Activation of CD25(+)CD4(+) regulatory T cells by oral antigen administration. Journal of immunology. 2001;167(8):4245-53.
15. Zhou L, Lopes JE, Chong MM, Ivanov, II, Min R, Victora GD, et al. TGF-beta-induced Foxp3 inhibits T(H)17 cell differentiation by antagonizing RORgammat function. Nature. 2008;453(7192):236-40.
16. Chiacchio T, Casetti R, Butera O, Vanini V, Carrara S, Girardi E, et al. Characterization of regulatory T cells identified as CD4(+)CD25(high)CD39(+) in patients with active tuberculosis. Clinical and experimental immunology. 2009;156(3):463-70.
17. de Cassan SC, Pathan AA, Sander CR, Minassian A, Rowland R, Hill AV, et al. Investigating the induction of vaccine-induced Th17 and regulatory T cells in healthy, Mycobacterium bovis BCG-immunized adults vaccinated with a new tuberculosis vaccine, MVA85A. Clinical and vaccine immunology : CVI. 2010;17(7):1066-73.
18. Dwyer KM, Hanidziar D, Putheti P, Hill PA, Pommey S, McRae JL, et al. Expression of CD39 by human peripheral blood CD4+ CD25+ T cells denotes a regulatory memory phenotype. American journal of transplantation : official journal of the American Society of Transplantation and the American Society of Transplant Surgeons. 2010;10(11):2410-20.

2. BRIEF SUMMARY OF THE NONCLINICAL & CLINICAL DATA

The effectivity of *Nyaditum resae®* has been tested in different doses, dosage regimens (three times a week, daily), duration of treatment (10, 13 and 14 days), and administrations (prophylactically and therapeutically), in 6-8 weeks-old female spf mice (CeHeB/FeJ strain), using the experimental animal model based on the Kramnik mouse and characterized by Marzo et al [Damaging role of neutrophilic infiltration in a mouse model of progressive tuberculosis. Marzo E, Vilaplana C, Tapia G, Diaz J, Garcia V, Cardona PJ. Tuberculosis. Available online 19 September 2013, http://dx.doi.org/10.1016/j.tube.2013.09.004) .

The animals were infected with different batches of *Mycobacterium tuberculosis* (*H37Rv Pasteur* strain) endovenously with doses from $2x10^4$ to $6.2x10^5$ viable bacilli. The evolution of the disease course is different depending on the exact dose and the batch of used of the *Mycobacterium tuberculosis* (*H37Rv Pasteur* strain).

Nyaditum resae® was administered according to the appropriate protocol, orally (through a nasogastric tube).

All the procedures were performed and approved by the Animal Experimentation Ethics Committee of the Hospital *Universitari Germans Trias i Pujol*(registered as B9900005) and by the Dept. d'Agricultura, Ramaderia, Pesca, Alimentació i Medi Natural of the Catalan Government, in accordance with current national and European Union legislation regarding the protection of experimental animals (Law 1997 of the Catalan Government; Spanish *Real Decreto* 1201/2005; and the European 86/609/CEE; 91/628/CEE; 92/65/CEE and 90/425/CEE). The animals were observed, weighted daily and followed-up until their conditions required an endpoint according to the current european laws of Ethics in Animal Experimentation.

The administration of *Nyaditum resae®* in different doses and dosages increased the survival of the animals in all cases, the difference with the control group being statistically significant in most cases. The results of each experiment performed are presented in the next pages.

The safety of *Nyaditum resae®* has been evaluated in all the experiments performed, in a total of 216 animals. Its tolerability has been tested by monitoring the following conditions of the

animals: weight, behaviour (isoleness and agressivity signs) and apparent good health (hair and skin), with the aim to detect any adverse effect attributable to the investigational product. No adverse effect has been detected in any animal to which *Nyaditum resae*® had been administered, and *Nyaditum resae*® was well tolerated in all cases.

The table below summarizes the experiments conducted with *Nyaditum resae*® and their results. Detailed data is attached in the next pages.

Type of Study	Test System	Infective Dose & batch	NR batch	NR doses	Dosage	Testing Facility	Number/Code of the experiment
1: Primary Pharmacodynamics: evaluation of the effect of NR on the survival after the treatment of experimental active TB (against relapse). 2. Safety Pharmacology: effect on the general conditions (weight, behaviour, general health aspect)	mice	$2{\cdot}10^5$ CFU/mL; *M.tuberculosis* Pasteur strain, batch 4	A#13	$3{\cdot}10^6$ CFU/mL (1:1000 dilution of a NR vial)	5 days a week (Monday-friday), total doses=10	Unitat de Tuberculosi Experimental (UTE). Fundació Institut Germans Trias i Pujol (Badalona, Catalonia, Spain)	nyada #18
1: Primary Pharmacodynamics: evaluation of the effect of NR on the survival (when administered prophylactically). 2. Safety Pharmacology: effect on the general conditions (weight, behaviour, general health aspect)	mice	$2{\cdot}10^5$ CFU/mL; *M.tuberculosis* Pasteur strain, batch 4	A#13	$3{\cdot}10^6$ CFU/mL (1:1000 dilution of a NR vial)) or $3{\cdot}10^8$ CFU/mL (1:10 dilution of a NR vial)).	3 days a week (Monday-Wednesday-friday), total doses= 13	Unitat de Tuberculosi Experimental (UTE). Fundació Institut Germans Trias i Pujol (Badalona, Catalonia, Spain)	nyada #16-prophyilaxis
1. Primary Pharmacodynamics: evaluation of the effect of NR on the survival (when administered therapeutically). 2. Safety Pharmacology: effect on the general conditions (weight, behaviour, general health aspect)	mice	$2{\cdot}10^5$ CFU/mL; *M.tuberculosis* Pasteur strain, batch 4	A#13	$3{\cdot}10^6$ CFU/mL (1:1000 dilution of a NR vial)) or $3{\cdot}10^8$ CFU/mL (1:10 dilution of a NR vial)).	5 days a week (Monday to Friday), total doses = 13	Unitat de Tuberculosi Experimental (UTE). Fundació Institut Germans Trias i Pujol (Badalona, Catalonia, Spain)	nyada #16-therapeutic
1: Primary Pharmacodynamics: evaluation of the effect of NR on the survival (when administered therapeutically). 2. Safety Pharmacology: effect on the general conditions (weight, behaviour, general health aspect)	mice	$2{\cdot}10^4$ CFU/mL; *M.tuberculosis Pasteur* strain, batch 4	A#13	$3{\cdot}10^7$ CFU/mL (1:100 dilution of a NR vial)	diàriament, dosis totals = 14	Unitat de Tuberculosi Experimental (UTE). Fundació Institut Germans Trias i Pujol (Badalona, Catalonia, Spain)	nyada #19
1: Primary Pharmacodynamics: evaluation of the effect of NR on the survival (when administered prophylactically or therapeutically). 2. Safety Pharmacology: effect on the general conditions (weight, behaviour, general health aspect)	mice	$6.2{\cdot}10^5$ CFU/mL; *M.tuberculosis Pasteur* strain, batch 4	A#13	$3{\cdot}10^7$ CFU/mL (1:100 dilution of a NR vial) or $3{\cdot}10^6$ CFU/mL (1:1000 dilution of a NR vial)	Daily. Total doses = 14.	Unitat de Tuberculosi Experimental (UTE). Fundació Institut Germans Trias i Pujol (Badalona, Catalonia, Spain)	nyada #21
1: Primary Pharmacodynamics: evaluation of the effect of NR on the survival (when administered therapeutically). 2. Safety Pharmacology: effect on the general conditions (weight, behaviour, general health aspect)	mice	amb $4{\cdot}10^4$ CFU/mL; *M.tuberculosis Pasteur* strain, batch 4	A#13	$3{\cdot}10^6$ CFU/mL (1:1000 dilution of a NR vial) or $3{\cdot}10^5$ CFU/mL (1:10000 dilution of a NR vial)	Daily. Total doses = 14.	Unitat de Tuberculosi Experimental (UTE). Fundació Institut Germans Trias i Pujol (Badalona, Catalonia, Spain)	nyada #23
1: Primary Pharmacodynamics: evaluation of the effect of NR on the survival (when administered therapeutically). 2. Safety Pharmacology: effect on the general conditions (weight, behaviour, general health aspect)	mice	amb $4{\cdot}10^4$ CFU/mL; *M.tuberculosis Pasteur* strain, batch 4	A#13	$3{\cdot}10^5$ CFU/mL (1:10000 dilution of a NR vial)	Daily. Total doses = 14.	Unitat de Tuberculosi Experimental (UTE). Fundació Institut Germans Trias i Pujol (Badalona, Catalonia, Spain)	nyada #25

The tolerability of *Nyaditum resae*® and its effect on the Treg cell populations will be tested in humans in a Clinical Trial (CT) to be launched in January 2014. Entitled as NYADATREG, this will be a double-blind, randomized, placebo-controlled Trial to investigate safety and immunogenicity of the new paraprobiotic *Nyaditum resae*® administered to adults with or without latent tuberculosis infection. The trial will be conducted in the Germans Trias i Pujol

Hospital and will evaluate the effect of two different doses (10^4 and 10^5 CFUs) of *Nyaditum resae®* in a population of *M. tuberculosis* infected (n=30) and healthy (n=30) people. This trial will be performed according to the current European laws of Ethics in Human Experimentation, and its protocol has been reviewed and approved by the Ethics Committee of the correspondent Hospital. A summary of the CT is attached to this document as Annex 1.

3. PRE-CLINICAL EXPERIMENTS CONDUCTED WITH *NYADITUM RESAE*®: PROTOCOL SUMMARY & RESULTS.

3.1. EXPERIMENT #1 (nyada #18)

OBJECTIVE:

To evaluate the possible effect of *Nyaditum resae® (NR)* on the survival after the standard treatment of experimental active tuberculosis (against relapse), when administered 5 days a week (Monday to Friday); total number of doses= 10.

M&M

The infection of the animals was performed with $2{\cdot}10^5$ CFU/mL of *M.tuberculosis* (Pasteur strain), batch 4. All experimental groups were treated with the standard antituberculous treatment (RIMSTAR®: Isoniazid, Ethambutol, Pyrazinamide and Rifampicine; adjusted to mice's weight) from week 2 post-infection. NR group received 0.3mL of *Nyaditum resae®,* corresponding to *a* $3{\cdot}10^6$ CFU/mL dose (1:1000 dilution of a NR vial).

Experimental Groups:

1. Control group: RIMSTAR®, no NR-treated (n=12)
2. Preinfection treatment group: RIMSTAR® + NR, 10 doses; 5 days a week (Monday to Friday). Treatment was initiated 72h after ending the RIMSTAR® treatment.

Conclusions:

1. Even if not being different in a statistically significant way, the NR-treated animals had an increased survival rate than those of the control group, thus it can be inferred that NR provides a better protection to the standard antituberculous treatment in terms of protection against relapse.

2. NR was well-tolerated.

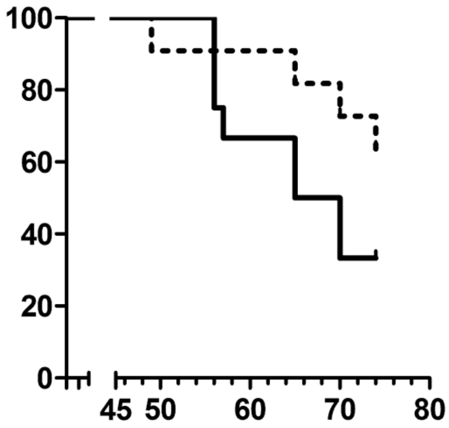

Species	Strain	Gender, total n and n per experimental group	Infective dose & batch	NR batch	NR batch	Administration route	Dosage	Results	Number/code assay
mice	C3HeB/FeJ	females, total n= 24, n per grup= 12	$2{\cdot}10^5$ CFU/mL; *M.tuberculosis* Pasteur strain, batch 4	A#13	$3{\cdot}10^6$ CFU/mL (1:1000 dilution of a NR vial)	orally	5 days a week (Monday-friday); total doses=10	1. Even if not being different in a statistically significant way, the NR-treated animals had an increased survival rate than those of the control group, thus it can be inferred that NR provides a better protection to the standard antituberculous treatment in terms of protection against relapse.<br>2. NR was well-tolerated.	nyada #18

3.2. EXPERIMENT #2 (nyada #16-prophylaxis)

OBJECTIVE:

To evaluate the possible prophylactic effect of *Nyaditum resae*® *(NR)* on the survival after two different doses of NR when administered three times a week (Monday-Wednesday-Friday); total number of doses= 13.

M&M

The infection of the animals was performed with $2{\cdot}10^5$ CFU/mL of *M.tuberculosis* (Pasteur strain), batch 4. NR group received 0.3mL of *Nyaditum resae*®, corresponding to a $3{\cdot}10^6$ CFU/mL dose (1:1000 dilution of a NR vial) or $3{\cdot}10^8$ CFU/mL dose (1:10 dilution of a NR vial).

Experimental Groups:

1. Control group: no treated (n=12)
2. Prophylactic group, a $3{\cdot}10^6$ CFU/mL dose : the infection was performed 24 hours after the last dose was administered (n=12).
3. Prophylactic group, a $3{\cdot}10^8$ CFU/mL dose : the infection was performed 24 hours after the last dose was administered (n=12).

Conclusions:

1. The NR-treated animals shown an increased survival rate than those of the control group, in a statistical significant way.

2. The experimental group receiving the lowest dose (more diluted) shown better results than the group receiving the highest dose (less diluted). This difference was not statistically significant.

3. NR was well-tolerated.

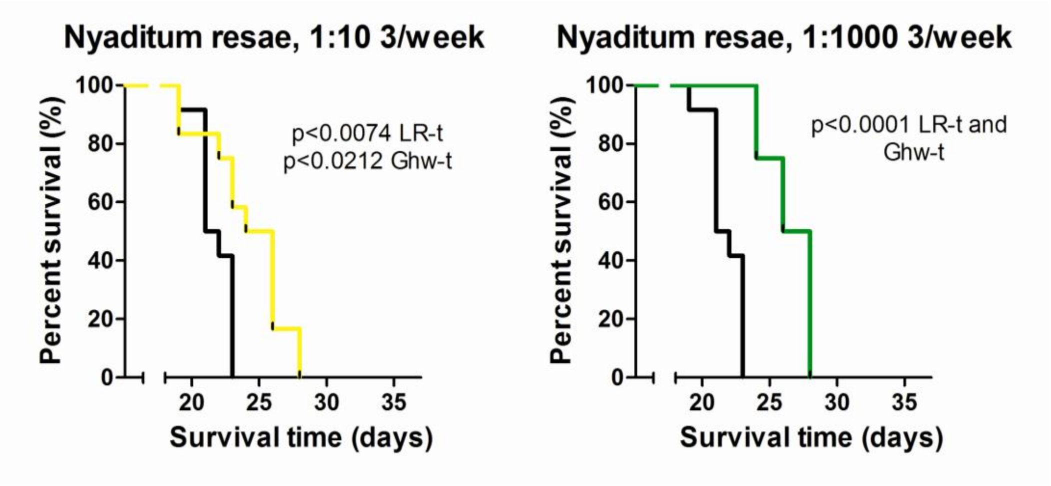

Species	Strain	Gender, total n and n per experimental group	Infective dose & batch	NR batch	NR batch	Administration route	Dosage	Results	Number/code assay
mice	C3HeB/FeJ	females, total n= 36, n per grup= 12	$2·10^5$ CFU/mL; *M.tuberculosis Pasteur* strain, batch 4	A#13	$3·10^6$ CFU/mL (1:1000 dilution of a NR vial) or $3·10^8$ CFU/mL (1:10 dilution of a NR vial).	orally	3 days a week (monday-Wednesday-friday); total doses = 13	1. The NR-treated animals shown an increased survival rate than those of the control group, in a statistical significant way.<br>2. The experimental group receiving the lowest dose (more diluted) shown better results than the group receiving the highest dose (less diluted). This difference was not statistically significant.<br>3. NR was well-tolerated.	nyada #16

3.3. EXPERIMENT #3 (nyada #16-therapeutic)

OBJECTIVE:

To evaluate the possible therapeutic effect of *Nyaditum resae® (NR)* on the survival after two different doses of NR when administered five times a week (Monday to Friday); total number of doses= 13.

M&M

The infection of the animals was performed with $2 \cdot 10^5$ CFU/mL of *M.tuberculosis* (Pasteur strain), batch 4. NR group received 0.3mL of *Nyaditum resae®,* corresponding to a $3 \cdot 10^6$ CFU/mL dose (1:1000 dilution of a NR vial) or $3 \cdot 10^8$ CFU/mL dose (1:10 dilution of a NR vial).

Experimental Groups:

1. Control group: no treated (n=12)
2. Therapeutic group, a $3 \cdot 10^6$ CFU/mL dose: treatment was started 3 days after infection (n=12).
3. Therapeutic group, a $3 \cdot 10^8$ CFU/mL dose: treatment was started 3 days after infection (n=12).

Conclusions:

1. The NR-treated animals shown an increased survival rate than those of the control group, in a statistical significant way.

2. The experimental group receiving the lowest dose (more diluted) shown better results than the group receiving the highest dose (less diluted). This difference was not statistically significant.

3. NR was well-tolerated.

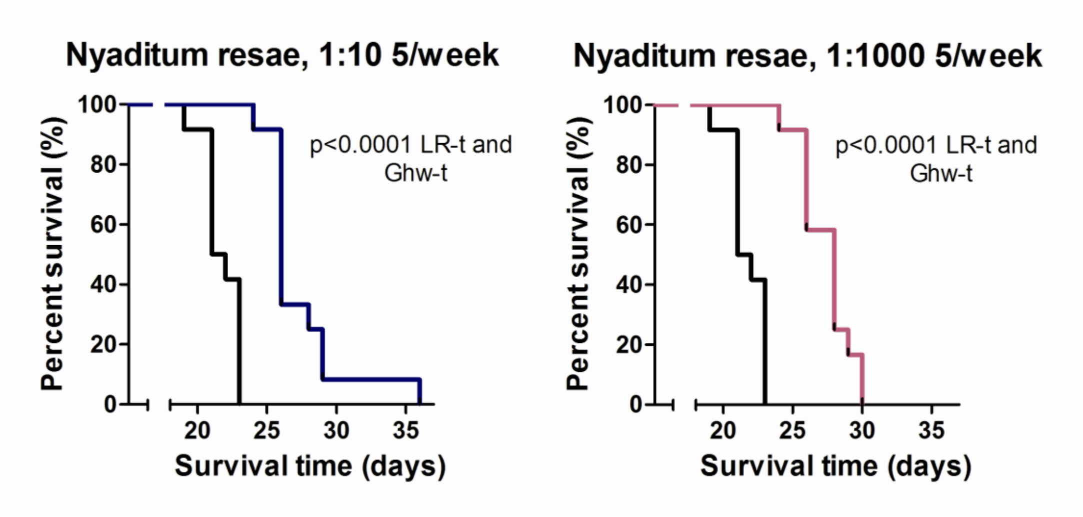

Species	Strain	Gender, total n and n per experimental group	Infective dose & batch	NR batch	NR batch	Administration route	Dosage	Results	Number/code assay
mice	C3HeB/FeJ	females, total n= 36, n per grup= 12	$2{\cdot}10^{5}$ CFU/mL; *M.tuberculosis Pasteur* strain, batch 4	A#13	$3{\cdot}10^{6}$ CFU/mL (1:1000 dilution of a NR vial) or $3{\cdot}10^{8}$ CFU/mL (1:10 dilution of a NR vial).	orally	5 days a week (Monday-friday); total doses = 13	1. The NR-treated animals shown an increased survival rate than those of the control group, in a statistical significant way.<br>2. The experimental group receiving the lowest dose (more diluted) shown better results than the group receiving the highest dose (less diluted). This difference was not statistically significant.<br>3. NR was well-tolerated.	nyada #16

3.4. EXPERIMENT #4 (nyada #19)

OBJECTIVE:

To evaluate the possible therapeutic effect of one dose of *Nyaditum resae® (NR)* on the survival when administered daily; total number of doses= 14.

M&M

The infection of the animals was performed with $2{\cdot}10^4$ CFU/mL of *M.tuberculosis* (Pasteur strain), batch 4. NR group received 0.3mL of *Nyaditum resae®,* corresponding to a $3{\cdot}10^7$ CFU/mL dose (1:100 dilution of a NR vial).

Grups experimentals:

1. Control group: no treated (n=12)
2. NR-treated group (n=12): treatment was started 5 days after infection.

Conclusions:

1. The NR-treated animals shown an increased survival rate than those of the control group, in a statistical significant way.

2. NR was well- tolerated.

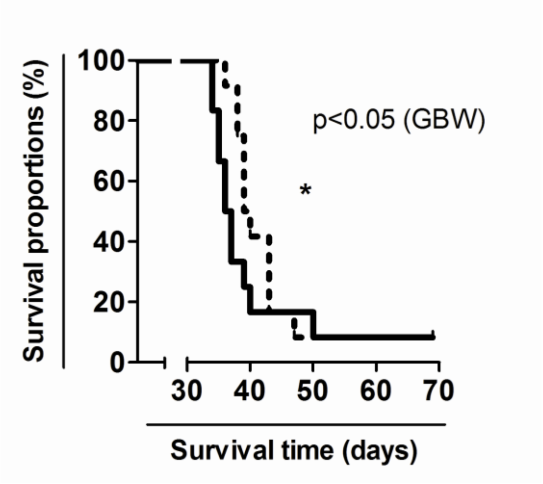

Species	Strain	Gender, total n and n per experimental group	Infective dose & batch	NR batch	NR batch	Administration route	Dosage	Results	Number/code assay
mice	C3HeB/FeJ	females, total n= 24, n per grup= 12	$2{\cdot}10^4$ CFU/mL; *M.tuberculosis Pasteur* strain, batch 4	A#13	$3{\cdot}10^7$ CFU/mL (1:100 dilution of a NR vial)	orally	daily; total doses = 14	1. The NR-treated animals shown an increased survival rate than those of the control group, in a statistical significant way. 2. NR was well- tolerated.	nyada #19

3.5. EXPERIMENT #5 (nyada #21)

OBJECTIVE:

To evaluate the possible prophylactic and therapeutic effect of two doses of *Nyaditum resae*® (NR) on the survival when administered daily; total number of doses= 14.

M&M

The infection of the animals was performed with $6.2 \cdot 10^5$ CFU/mL of *M.tuberculosis* (Pasteur strain), batch 4. The NR-treated animals received 0.3mL of *Nyaditum resae®,* corresponding to a $3 \cdot 10^7$ CFU/mL dose (1:100 dilution of a NR vial) or $3 \cdot 10^6$ CFU/mL dose (1:1000 dilution of a NR vial) according to the corresponding experimental group.

Experimental Groups:

1. Control group: no treated (n=12)
2. Prophylactic group, $3 \cdot 10^7$ CFU/mL dose (n=12). The infection was performed 1 week after the last dose of NR.
3. Prophylactic group, $3 \cdot 10^6$ CFU/mL dose (n=12). The infection was performed 1 week after the last dose of NR.
4. Therapeutic group, $3 \cdot 10^7$ CFU/mL dose (n=12). The treatment was started 1 week after the last dose of NR.
5. Therapeutic group, $3 \cdot 10^6$ CFU/mL dose (n=12). The treatment was started 1 week after the last dose of NR.

Conclusions:

1. The NR-treated animals (NR administered either prophylactically or therapeutically) shown an increased survival rate than those of the control group, in a statistical significant way.

2. NR was well-tolerated.

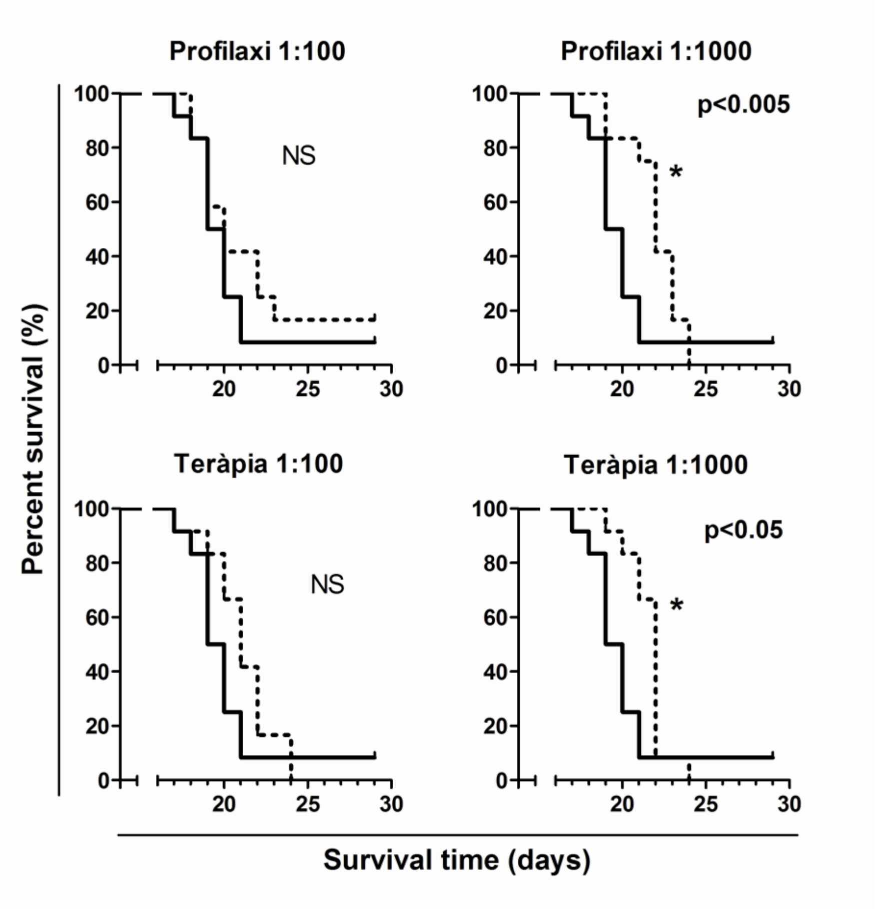

Species	Strain	Gender, total n and n per experimental group	Infective dose & batch	NR batch	NR batch	Administration route	Dosage	Results	Number/code assay
mice	C3HeB/FeJ	females, total n= 36, n per grup= 12	$6.2 \cdot 10^5$ CFU/mL; *M.tuberculosis Pasteur* strain, batch 4	A#13	a $3 \cdot 10^7$ CFU/mL (1:100 dilution of a NR vial) or $3 \cdot 10^6$ CFU/mL (1:1000 dilution of a NR vial)	orally	daily; total doses = 14	1. The NR-treated animals (NR administered either prophylactically or therapeutically) shown an increased survival rate than those of the control group, in a statistical significant way. 2. NR was well-tolerated.	nyada #21

3.6. EXPERIMENT #6 (nyada #23)

OBJECTIVE:

To evaluate the possible therapeutic effect of two doses of NR (($3{\cdot}10^6$ CFU/mL (1:100 dilution of a NR vial) o $3{\cdot}10^5$ CFU/mL (1:1000 dilution of a NR vial) on the survival when administered daily; total number of doses= 14.

M&M

The infection of the animals was performed with $4{\cdot}10^4$ CFU/mL of *M.tuberculosis* (Pasteur strain), batch 4. The NR-treated animals received 0.3mL of *Nyaditum resae®,* corresponding to a $3{\cdot}10^6$ CFU/mL dose (1:1000 dilution of a NR vial) or $3{\cdot}10^5$ CFU/mL dose (1:10000 dilution of a NR vial) according to the corresponding experimental group.

Grups experimentals:

1. Grup control: sense tractament (n=12)
2. Grup tractat amb NR (dosi $3{\cdot}10^6$ CFU/mL (dilució 1:1000 d'un vial de NR)(n=12). El tractament es va començar el mateix dia de la infecció.
3. Grup tractat amb NR (dosi $3{\cdot}10^5$ CFU/mL (dilució 1:10000 d'un vial de NR)(n=12). El tractament es va començar el mateix dia de la infecció.

Experimental Groups:

1. Control group: no treated (n=12)
2. NR group, $3{\cdot}10^6$ CFU/mL dose (n=12). Treatment was started the same day the infection was performed.
3. NR group, $3{\cdot}10^5$ CFU/mL dose (n=12). Treatment was started the same day the infection was performed.

Conclusions:

1. The NR-treated animals shown an increased survival rate than those of the control group (not-statistical significant difference).

2. NR was well-tolerated.

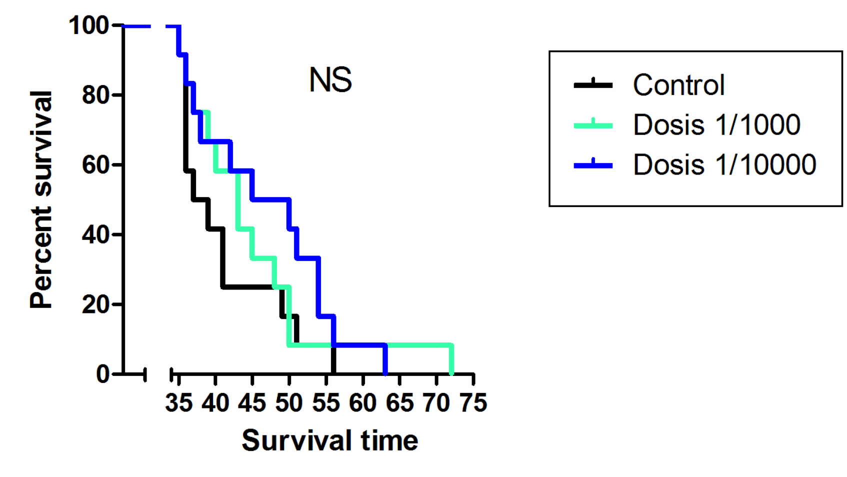

Species	Strain	Gender, total n and n per experimental group	Infective dose & batch	NR batch	NR batch	Administration route	Dosage	Results	Number/code assay
mice	C3HeB/FeJ	females, total n= 36, n per grup= 12	amb $4{\cdot}10^4$ CFU/mL; *M.tuberculosis Pasteur* strain, batch 4	A#13	$3{\cdot}10^6$ CFU/mL (1:1000 dilution of a NR vial) or $3{\cdot}10^5$ CFU/mL (1:10000 dilution of a NR vial)	orally	daily; total doses = 14	1. The NR-treated animals shown an increased survival rate than those of the control group (not-statistical significant difference).<br>2. NR was well-tolerated.	nyada #23

3.7. EXPERIMENT #7 (nyada #25)

OBJECTIVE:

To evaluate the possible effect of one dose of NR (($3{\cdot}10^5$ CFU/mL (1:10000 dilution of a NR vial) on the survival when administered daily; total number of doses= 14.

M&M

The infection of the animals was performed with $4{\cdot}10^4$ CFU/mL of *M.tuberculosis* (Pasteur strain), batch 4. The NR-treated animals received 0.3mL of *Nyaditum resae®,* corresponding to $3{\cdot}10^5$ CFU/mL dose (1:10000 dilution of a NR vial).

Experimental Groups:

1. Control group: no treated (n=12)
2. NR group, dosi $3{\cdot}10^5$ CFU/mL dose (n=12). Treatment was started the same day the infection was performed.

Conclusions:

1. The NR-treated animals shown an increased survival rate than those of the control group (not-statistical significant difference).

2. NR was well-tolerated.

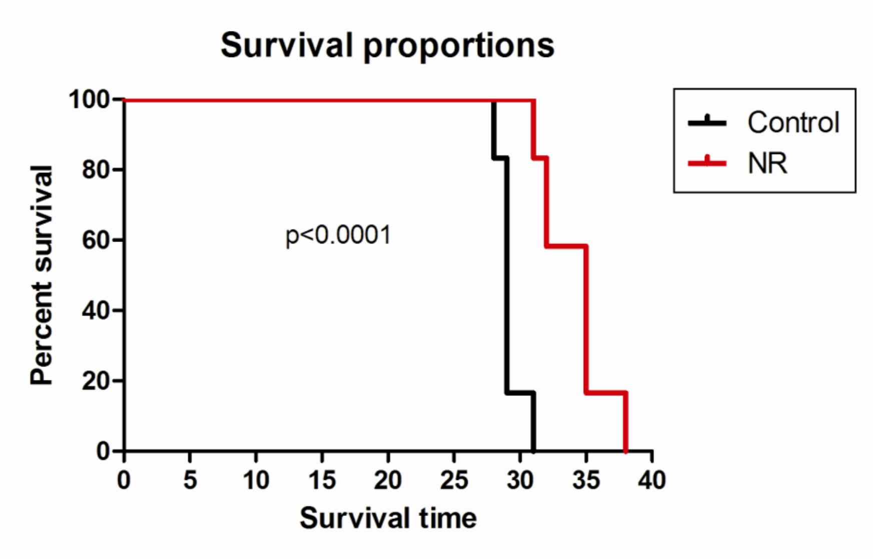

Species	Strain	Gender, total n and n per experimental group	Infective dose & batch	NR batch	NR batch	Administration route	Dosage	Results	Number/code assay
mice	C3HeB/FeJ	females, total n= 24, n per grup= 12	amb $4{\cdot}10^4$ CFU/mL; *M.tuberculosis Pasteur* strain, batch 4	A#13	3·105 CFU/mL (1:10000 dilution of a NR vial)	orally	daily; total doses = 14	1. The NR-treated animals shown an increased survival rate than those of the control group (not-statistical significant difference).<br>2. NR was well-tolerated.	nyada #25

ANNEX 1:

PROTOCOL OF THE *Nyaditum resae*® CT, NYADATREG-1. SYNOPSIS.

Hypothesis

The oral administration of a low-dose (10^4 or 10^5 CFU) of *Nyaditum resae®* during 14 days is well-tolerated and generates a specific memory regulatory T cell population.

Objectives

1.- Evaluation of the tolerability of two doses (10^4 and 10^5 CFU/mL) of the probiotic *Nyaditum resae®* when compared to placebo, in adults with or without Latent Tuberculosis Infection (LTBI).

2.- Evaluation of the immunogeniciy by means of the generation of specific memory regulatory T cells one week after the oral administration of the probiotic *Nyaditum resae® during 14 days* when compared to placebo.

Description of the treatment and definition of the exposition

The subjects will be stratified according to LTBI: LTBI positive and LTBI negative.

There will be 3 treatment arms:

- Group A: *Nyaditum resae®* 10^4 UFC
- Group B: *Nyaditum resae®* 10^5 UFC
- Group C: Placebo

The Placebo contain the same excipients than *Nyaditum resae®.*

Once aleatorized, the subjects will receive a total of 14 capsules of *Nyaditum resae®* or placebo according to their experimental group. They will take orally one capsule per day, during 2 weeks. The participants will be followed-up during a total of 6 weeks after starting the treatment.

A total of 60 subjects (30 LTBI positive and 30 LTBI negative) will be aleatorized in one of the 6 treatment groups, as presented in the following table:

Group		n of subjects and LTBI status	Composition	Dose	Route
1	A1	10 LTBI pos	Nyaditum resae® 10^4	The subjects will receive 14 capsules of Nyaditum resae® 10^4 UFCs	Oral
2	A2	10 LTBI neg			
3	B1	10 LTBI pos	Nyaditum resae® 10^5	The subjects will receive 14 capsules of Nyaditum resae® 10^5 UFCs	Oral
4	B2	10 LTBI neg			
5	C1	10 LTBI pos	Placebo	The subjects will receive 14 capsules of Placebo	Oral
6	C2	10 LTBI neg			

VARIABLES AND MEASUREMENTS

Study variables

Safety variables:

- Local tolerability (gastrointestinal)
- General tolerability

Immunogenicity variables:

- Generation of memory Treg cells

Measurements

At baseline: Physical examination and Demographic data (including Mantoux and thorax Rx if Mantoux positive)

Laboratory Analysis: hematology parameters; biochemistry parameters; pregnancy test (if women); microbiology parameters (HIV serology); Immunogenicity parameters (cito and chemokine profile, PPD-specific memory Treg cell populations)

A diary will be collected for each subject included in the trial, where adherence and tolerability data will be recorded (date and hour of the treatment intake, abdominal pain, deposition history, nausea, other serious adverse effects).

All the measurements will be performed according to the following plan.

	Selection		Pre adm.	Adm.	Follow-up		
Visit	Selection		Baseline		W1	W2	W6
Day	-28	-25	0		7	15	42
Week	-4	-4	0		1	2	6
Window (days)	±14		0		±3	±3	±3
Informed Consent	X						
Inclusion/Exclusion Criteria	X		X				
Medical History	X						
HIV test	X						
Mantoux	X[A]	X[B]					
Radiodiagnosis (thorax Rx)		X[C]					
Pregnant test in urine	X		X				X
Randomization			X				
Treatment Administration				X			
Physical examination	X		X		X	X	X
Sampling for Biochemical and Hematological parameters	X		X[D]		X	X	X
Immunogenicity sampling			X		X	X	X
Subject Diary				X	X	X	X[E]
Concomitant Medication	X		X		X	X	X
Adverse effects	X			X	X	X	X

[A] *A positive test during the last 5 years or negative test during the last 6 months will be accepted.*
[B] *Visit to read the Mantoux result.*
[C] *If the Mantoux is positive.*
[D] No needed to be repeated if the selection visit has been done in the previous 15 days.
[E] *Subject Dairy submission.*
