# Supplementary material for: Pilot, double-blind, randomized, placebo-controlled clinical trial of the supplement food Nyaditum resae® in adults with or without latent TB infection: Safety and immunogenicity
Source: PLoS One. 2017 Feb 9;12(2):e0171294. doi: 10.1371/journal.pone.0171294 (PMC5300153; doi:10.1371/journal.pone.0171294)
Supplement: S5 File — (PDF) [file pone.0171294.s005.pdf]

PARTICIPANT INFORMATION SHEET

Double-blind, randomized, masked, placebo-controlled, Clinical Trial to investigate the tolerability and immunogenicity of the probiotic Nyaditum resae® administered to adults with or without latent tuberculosis infection.

This data sheet may contain terms that you do not understand. Do not hesitate to ask your doctor or the attending personal any questions that you may have.

INTRODUCTION

You are kindly requested to take part in a medical-scientific study. This study was approved by the Clinical Research Ethics Committee of the Hospital Germans Trias i Pujol.

Our intention is just to provide you with the correct and necessary information to help you consider and decide if you want to participate or not. For this, we are asking you to read this information carefully. The study's doctor will clarify any doubts you may have after reading the explanation. You can also discuss it with any person you deem appropriate such as relatives, friends and your regular physician to help you take a decision.

VOLUNTARY PARTICIPATION

You should know that your participation in this is study is voluntary and that you can decide to not participate or change your decision and withdraw the informed consent at any time without causing any kind of damage to you

GENERAL DESCRIPTION OF THE STUDY

The incidence of tuberculosis is still a problem of great magnitude. Each year 1.5 million people die because of tuberculosis; there are 10 million cases and 100 million of new infected cases. In addition, there is a new problem of multi –resistance that remains prevalent: about 700,000 patients, a figure that is growing annually with 100,000 people. The prevention of tuberculosis is currently very difficult since this is a disease caused by an airborne bacillus: there is no a risk factor to be infected and there is still no prophylactic vaccine to prevent the infection. One of the most characteristic aspects of tuberculosis is that most of people (90-95%) without alterations in the immunity do not develop the disease after the infection. As for people who develop the disease it is still unknown why they develop it.

A group of researchers at the Institut Gemans Trias i Pujol recently found a mechanism that explain this evolution. In summary, what happens is that the immunity of certain people generates an excessive immune response against the bacteria, which ends up creating massive destruction of the tissue around the bacteria and this causes the characteristic lesion of tuberculosis: the tuberculous cavity.

This group of researchers devised the method to "re-educate" the immune system against the bacilli to do it less aggressive. And they did it using two instruments. The first one, an environmental mycobacterium, in other words, a bacillus from the family of mycobacteria but that usually lives in the water we drink, so to a greater or lesser extent we already have in our intestinal flora. The second one, inducing a *tolerant response* as it is done when you ingest food. To induce a tolerant response, we administrate low and repeated doses (non homeopathic) of the product so that the immune system of the gastrointestinal tract "become accustomed" to their presence. Thus, when it becomes to find the product, the immune system reacts in a mild and balanced way, avoiding excessive responses. An example of this is the fact that our immune system "is used" to food proteins and do not generate rejection responses when he finds them in the intestinal mucosa.

And this is Nyaditum resae®, a preparation in a capsule form containing an heat-killed environmental mycobacterium and, therefore, can generate a cross –immunity with the tuberculosis bacillus. In small and repetitive dosages, we manage to generate a tolerant response, which is what happens when there is an infection of *Mycobacterium tuberculosis*, so that it is not possible to trigger the excessive inflammatory reaction that causes tuberculosis disease.

STUDY METHODS

Initially there will be a selection process in which, in addition to realize the medical record, physical examination and vital signs, a tuberculin skin test will be done. If the participant has already done this test, he will be accepted if it is positive less than 5 years and if it is negative less than 6 months. In other cases, the test will be repeated.

The tuberculin skin test is based on the subcutaneous administration of an extract of the tubercle bacillus to test whether a response of the immune system is generated demonstrating that the person is infected. The test is read 3 days after the administration and if it is positive, a chest X-ray will be necessary to discard you do not suffer tuberculosis. If the tuberculin skin test is positive and discarded tuberculosis, you will be considered as an infected person but not suffering the disease. You can continue participating in the study but your doctor will show the instructions to follow.

If the chest X –ray shows the presence of tuberculosis or anything else clinically relevant you will be referred to emergency and you can not participate in the study.

If you are selected, you will start the treatment with Nyaditum resae® or placebo (a substance with no pharmacologic effect) and you will be randomly assigned one of the different study groups (that means you will be assigned one group or another at random):

- Group A: Nyaditum resae® 10^4 UFC (low dose)
- Group B: Nyaditum resae® 10^5 (high dose)
- Group C: Placebo

This is a double –blind study which means that neither you nor your doctor will know in which group you have been included.

In all cases, the treatment will be administrated orally and consist of 14 capsules which will be administrated daily when having breakfast.

It is very important to take all the capsules of the treatment during the period of the study. In addition, if you are taking or have taken during the last two weeks any other medication, you have to mention it to your doctor in order to register it in your medical record.

The estimated duration of the study will be 6 weeks. The first day of the treatment a visit will be done, another visit one week after the start of the treatment and finally two more visits, 2 and 6 weeks after the start of the treatment.

In each visit blood samples will be collected (with a maximum of 20 mL per visit) in order to determine the immune response generated because of the treatment and to perform the product safety control of both parameters, haematological and biochemical. Likewise, you will be asked for any pain you might have had. Also, your vital signs will be taken and a physical examination will be performed again. We ask for fill out a diary during the first 4 weeks of treatment to know if you have taken all the treatment and also to know if you have had any symptoms.

The immunogenicity study and the profile of cytokines will be made at the Experimental Tuberculosis Unit. The rest of analysis will be made at the laboratories of the Hospital Germans Trias i Pujol

The results will be introduced in a database and they will be used only for scientific purposes. His management will be carried out only by a qualified person.

POTENTIAL SIDE EFFECTS

There are no data available about the safety of the probiotic in humans. The only potential adverse event expected, due to the oral administration, is a mild alteration of the bowel transit.

The collection of blood samples can cause local pain or haematoma as because of the puncture.

The puncture of the tuberculin skin test can cause local transient discomfort.

For women:
If you are pregnant or think you might be pregnant you are not able to participate in the study. That's why before entering the study it is necessary to make a pregnancy test to prove that is negative. During the study you should not get pregnant, and for that reason it is recommended to use a contraceptive method. Even though a pair of pregnancy test will be made (during and at the end of the study).

It is not allowed to participate to nursing women too.

INSURANCE

The study's sponsor has an insurance policy as per current law and it would provide a compensation and indemnity for any health issues or injuries resulting from your participation in this study.

CONFIDENTIALITY

The information derived from this study will be sent to the sponsor. The data will be coded to preserve your identity, according to the Spanish Organic Law 15/1999 from December 13th, devoted to personal data protection. According to this law you have the right to access, change, oppose and cancel your data. To do any of these you must contact your study's physician. You can rest assure that your clinical history information will be strictly confidential and your identity will be kept anonymous. Only the study's physician or collaborators will be able to match those data with you will not be revealed to anybody except when required by medical emergencies or legal requests. The following will have Access to your clinical history: the sponsor´s authorized representative, the Ethics Committee or the relevant Health Authority, in order to review the information. Your identity will remain confidential even if some results of the study are published.

The study's data sent to third parties and to other countries will never contain information that could directly identify you such as, name and last name, address, Social Security number, etc. If this information was revealed, it would be for the same purposes related to the study mentioned before and granting the data protection based on the laws in our country.

ECONOMIC COMPENSATION

The study's sponsor is the responsible person of the funding management. To perform this study, the sponsor has signed a contract with the institute and with the study physician.

Your participation in this study do not represent any expense for you. In fact, you will be financially compensated for the time spent and the inconvenience it caused. The number of visits in this study can vary from 5 to 7. The financial compensation for your participation will be 50 euros for each day you have to go to the hospital. The payment will be made through bank transfer at the end of the study, this is why we will need you full name, address, ID number and bank account number.

OTHER RELEVANT INFORMATION

Any new information in relation with the treatment used in this study and that may affect you availability to participate in the study will be communicated by your physician as soon as possible.

If you decide to withdraw the consent to participate in this study, no new data will be added to the database and you may require the destruction of all indentificable samples to avoid performing new analysis.

You should also know that you may be excluded from the study if the sponsor or researchers of the study feel it appropriate, whether for security reasons (any adverse event due to administration the medication of the study) or because they consider you are not fulfilling the requirements of the study. In any case, you will receive an appropriate explanation about the withdrawal reason.

By signing the informed consent attached you agree to follow all the procedures of the study that have been exposed

POTENTIAL BENEFITS OF THE STUDY

The global goal of this study is to know the effect of Nyaditum resae ® on the immunity and the protection against the development of tuberculosis. In any case, your participation in this study may be of great help to understand how this product works.

If you have any doubt, you must contact the responsible physician of the study, Dr. Eva Montane, in the following phone numbers 93 497 88 65 or 93 497 84 88 (UPIC).
