# Supplementary material for: Pilot, double-blind, randomized, placebo-controlled clinical trial of the supplement food Nyaditum resae® in adults with or without latent TB infection: Safety and immunogenicity
Source: PLoS One. 2017 Feb 9;12(2):e0171294. doi: 10.1371/journal.pone.0171294 (PMC5300153; doi:10.1371/journal.pone.0171294)
Supplement: S6 File — (PDF) [file pone.0171294.s006.pdf]

INFORMED CONSENT FOR PARTICIPANTS

Double-blind, randomized, masked, placebo-controlled, Clinical Trial to investigate the tolerability and immunogenicity of the probiotic Nyaditum resae® administered to adults with or without latent tuberculosis infection.

Study code: NYADATREG

I, ____
(participant surname and name)

I have read the information that it has been delivered to me
I could ask questions about the study
I have received enough information about the study

I talked to ____
(researcher's name, physician)

I understand that my participation is voluntary
I understand that I can withdraw from the study:

1. Whenever I want
2. Without explanations
3. Without consequences for my medical care

I give my consent to participate in this study and I consent to accessing and using the data related to the study under the conditions mentioned in the information sheet.

______________________________ ______________________________

(Participant's signature and date) (Researcher's signature and date)

This document will be signed in duplicate; one copy goes to the researcher and another one to the participant.
