# Supplementary material for: Pilot, double-blind, randomized, placebo-controlled clinical trial of the supplement food Nyaditum resae® in adults with or without latent TB infection: Safety and immunogenicity
Source: PLoS One. 2017 Feb 9;12(2):e0171294. doi: 10.1371/journal.pone.0171294 (PMC5300153; doi:10.1371/journal.pone.0171294)
Supplement: S7 File — (PDF) [file pone.0171294.s007.pdf]

MANREMYC

Manresana de Micobacteriologia sl.

MEDICACIÓN

- Registre cualquier medicación que tome distinta de la habitual.
- Incluya la dosis diaria total
- Cuando el tratamiento dure varios días, anote la fecha de inicio y final y la dosis diaria total.

FECHA	MEDICACIÓN (Nombre y dosis)

MANREMYC

Manresana de Micobacteriologia sl.

DIARIO DEL PARTICIPANTE
PROTOCOLO NYADATREG

ID Paciente: ________

PERSONAS DE CONTACTO

Dra. Ana Mª Barriocanal Barriocanal
Dra. Eva Montané Esteva
Servicio de Farmacología Clínica
Hospital Universitari Germans Trias i Pujol
Telf. 93 497 88 65 / 93 497 84 88

INFORAMCIÓN PARA RELLENAR EL DIARIO

- Utilice bolígrafo
- Rellene todas las casillas (aunque no haya tenido ningún síntoma)
- En caso de presentar alguna reacció grado 3 contacte con el equipo investigador
- Devuelva el diario en su próxima visita

Semana ________

**Fecha de la toma**								
**Hora de la toma**								
**Deposiciones (Nº/día)**								
**Náuseas (0–3)**								
**Vómitos (0-3)**								
**Dolor abdominal (0-3)**								
**Otros**								

0= Normal
1= Leve. Síntoma que causa una interferencia nula o mínima en las actividades funcionales.
2= Moderado. Síntoma que causa más que una interferencia mínima en las actividades funcionales.
3= Intenso. Síntoma que causa incapacidad para realizar actividades funcionales.

MANREMYC
Manresana de Micobacteriologia sl.

Semana ________

**Fecha de la toma**								
**Hora de la toma**								
**Deposiciones (Nº/día)**								
**Náuseas (0–3)**								
**Vómitos (0-3)**								
**Dolor abdominal (0-3)**								
**Otros**								

0= Normal
1= Leve. Síntoma que causa una interferencia nula o mínima en las actividades funcionales.
2= Moderado. Síntoma que causa más que una interferencia mínima en las actividades funcionales.
3= Intenso. Síntoma que causa incapacidad para realizar actividades funcionales.
