# Supplementary figures and images for: Pilot, double-blind, randomized, placebo-controlled clinical trial of the supplement food Nyaditum resae® in adults with or without latent TB infection: Safety and immunogenicity
Source: PLoS One. 2017 Feb 9;12(2):e0171294. doi: 10.1371/journal.pone.0171294 (PMC5300153; doi:10.1371/journal.pone.0171294)

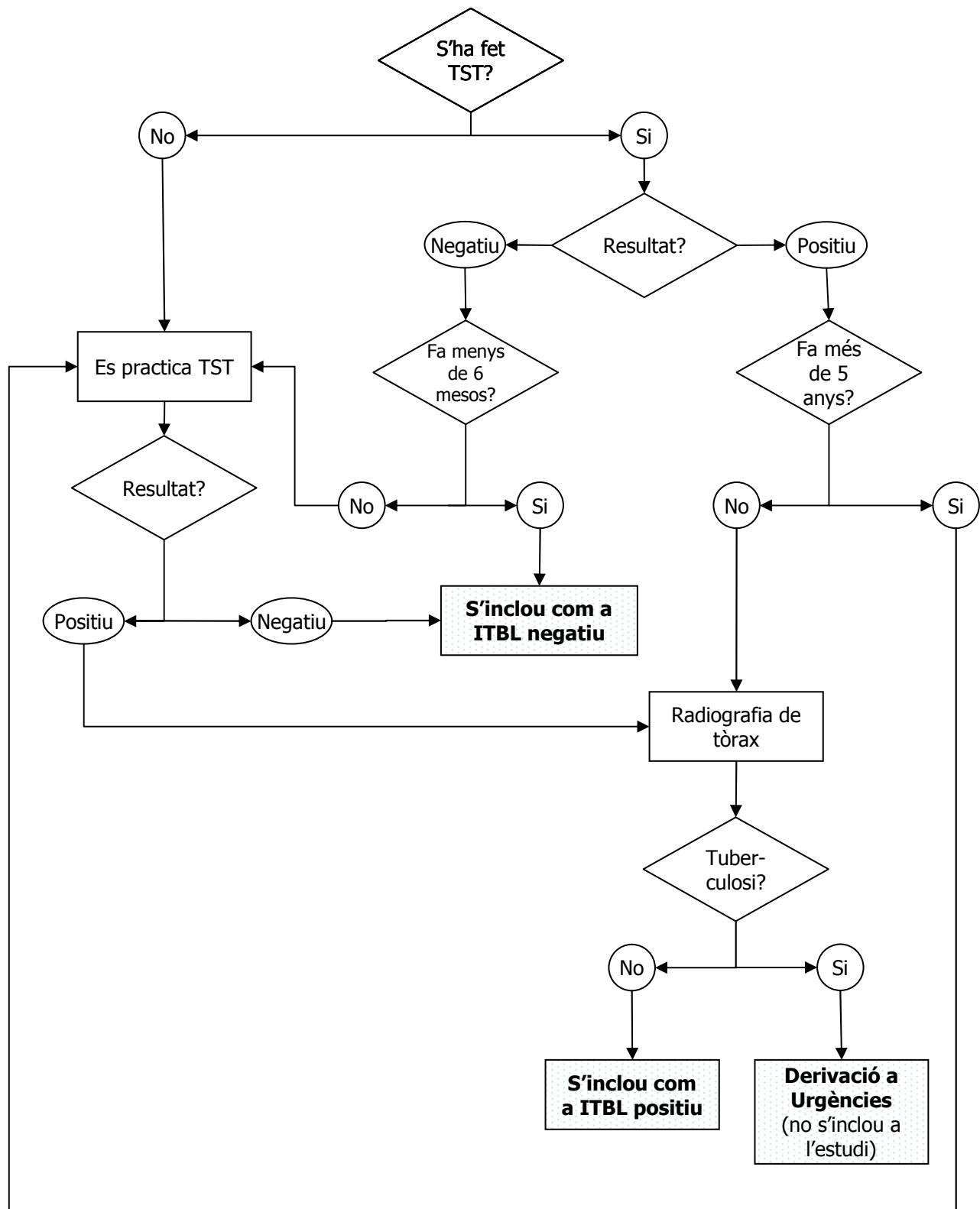

Supplement: S8 File — (PDF) [file pone.0171294.s008.pdf]
